# Development of a dynamical model to enhance understanding of epidemiology of schistosomiasis in school-aged-children

**DOI:** 10.1038/s41598-025-32664-w

**Published:** 2025-12-22

**Authors:** Amber L. Reed, Sekeleghe A. Kayuni, Janelisa Musaya, J. Russell Stothard, Claudio Fronterre, Chris Jewell

**Affiliations:** 1 Lancaster Medical School, Lancaster University, Bailrigg House, Bailrigg, Lancaster, LA1 4YE UK; 2 Tropical Disease Biology, Liverpool School of Tropical Medicine, Pembroke PI, Liverpool, L3 5QA UK; 3 Malawi Liverpool Wellcome Trust Programme of Clinical Tropical Research, Queen Elizabeth Central Hospital, College of Medicine, P.O.Box 30096, Blantyre, Malawi; 4 Department of Applied Health Sciences, School of Health Sciences, College of Medicine and Health, University of Birmingham, Birmingham, UK; 5 Mathematics and Statistics, Lancaster University, Bailrigg House, Bailrigg, Lancaster, LA1 4YE UK

**Keywords:** Schistosomiasis, *Biomphalaria*, *Bulinus*, Transmission dynamics, Optimisation, Diseases, Mathematics and computing

## Abstract

School-aged-children are most vulnerable to schistosomiasis as exemplified by age-prevalence profiles although current understanding of these patterns needs improvement. Using epidemiological data from the southern shoreline of Lake Malawi, we investigated the dynamics of *Schistosoma* transmission and the main determinants of *Schistosoma* infection risk using a transmission dynamic model, considering urogenital and intestinal schistosomiasis respectively. Specifically, we assessed whether the proximity of primary schools to the immediate shoreline was a major geospatial and epidemiological determinant. Cross-sectional parasitology and malacological data previously collected and analysed was used, including age-infection profiles and interpolated predicted snail distributions for the southern part of Lake Malawi shoreline. A disease SEIRS ordinary differential equation model was created, and an observation prevalence model was formed using a binomial sampling distribution using the already published dataset. An optimisation using L-BFGS-B algorithm with upper/lower bounded box constraints was carried out to calibrate the model to find the best parameter values for each infection state transition given the disease model and dataset. The aim was to recapture the age-structure dynamics shown in the observation model representing the already published age-infection profiles. Concerning intestinal schistosomiasis, the best model for *Biomphalaria* sp. was the use of a single transmission rate for all the school’s and no spatial effect. By contrast, for urogenital schistosomiasis, the best model for *Bulinus* spp. was found when using an independent transmission rate for each school and no spatial effect. There was some evidence that we were able to capture the age-structured dynamics of infection in SAC despite the expected outcome differing to statistical output due to sparse data. Within our study area, we found there was no significant effect on SAC exposure to *Schistosoma* infection risk based on school distance from the shoreline. Further, there was heterogeneity between schools in transmission rates estimated, although these did not have significantly different confidence intervals. However, schools considered in our study were all relatively close to cercaria infested shorelines. Further studies using a longitudinal cohort study could improve understanding of *Schistosoma* infection dynamics and allow for improved control method application.

**Supplementary Information:**

The online version contains supplementary material available at 10.1038/s41598-025-32664-w.

## Introduction

Schistosomiasis is a water-borne neglected tropical disease caused by the trematode worm *Schistosoma*, resulting in two forms of the disease, intestinal schistosomiasis (IS) caused by *S. mansoni* and urogenital schistosomiasis (UGS) caused by *S. haematobium* infections^1^. To complete their lifecycle, these species of *Schistosoma* require the presence of aquatic snail intermediate hosts, *Biomphalaria* sp. and *Bulinus* spp., respectively. Thus, exposure to the snail habitats is a known major risk factor for human *Schistosoma* infection^2^. School-aged-children (SAC) are known to be particularly vulnerable to schistosomiasis, with signs and symptoms of infection including malnutrition, anaemia, and neurological and developmental delays caused by the accumulation of trapped eggs causing tissue inflammation^3^. Children are thought to be first infected soon after birth upon freshwater contact(s) with prevalence increasing with cumulative parasite exposure(s) up to adolescence, although the age-profiles are known to oscillate over time due to many undefined factors^2^.

There are many known risk factors for *Schistosoma* infection transmission including repeated water contact, type of water use, animal contact, age and treatment^2,4–6^ Recent studies have reported an increase in transmission of *Schistosoma* infection through a changing ecological environment in the lake, an increasing human population and reduced molluscivore fish in the lake. This has created an increase in snail populations and new at-risk locations for water contact^7^. Changes in human behaviour affect age-dependent exposure, especially among SAC who are known for frequent water contact^7–9^. Reitzug et al*.*^9^, reported its importance in human exposure behaviour in driving *Schistosoma* transmission^9^. Other factors such as schistosomiasis infection immunity, formally known as resistance to reinfection, is still equivocal and being researched. Partially protective immunity in people developing over long repeated exposure to *Schistosoma* infection is much debated, alongside its interaction with age-dependent exposure^10^. Currently studies have reported some partial protection building up in individuals over time^10–13^. Most SAC, however, are considered to have very low to no acquired protective immunity allowing for reinfection(s) to occur. This is possibly shown by the Reed et al*.*^4^ study where the age profiles of SAC increase up to 11 years before decreasing there afterwards, although this is conjecture and requires more investigation^4,14^.

People are more likely to make use of water facilities close to where they live and if that water facility happens to be a *Schistosoma* infection transmission site then the risk is likely to be higher for these people than people living further away leading to a higher need for control application in communities closer to these high-risk areas^15^. Further, Madsen et al*.*^16^ reported higher *Schistosoma* transmission in shoreline villages compared to in-land villages^16^. Furthermore, other studies have also found the proximity to the lake to increase *Schistosoma* transmission risk^17,18^. Ecological niche mapping and fine-scale malacological mapping of areas have previously been used as a technique to try to measure exposure risk dependent on location; these studies find that there is localised variation in areas with different biological effects impacting the transmission for each focal location^19,20^. Other studies have researched water-contact related activities and association with exposure risk^9^. However, there is a lack of knowledge of how to measure SAC age-dependent exposure and the associated *Schistosoma* transmission risk, which is limiting appropriate application of control methods^4^. The difficulty of measuring this exposure could be due to multiple factors, for example, immunity, age of child, water exposure patterns and treatment^21^. Currently prevalence among SAC is measured at survey school locations using parasitological methods to measure prevalence and intensity of infection^22^. Further as mentioned before, age prevalence profiles among SAC have been studied along the southern part of the Lake Malawi shoreline and reported to oscillate over time^4^.

Mathematical modelling can allow us to model the spread of disease and transmission dynamics to inform policy decision makers for intervention programmes. Many mathematical modelling studies have been carried out over the years to aid control programmes^23–26^. Both Nelson & Macdonald carried out pioneering work on transmission dynamics of *Schistosoma* infection^27–29^. Anderson et al*.*^21^ recently reviewed transmission models and control of *Schistosoma* infection by Mass Drug Administration (MDA). The World Health Organisation (WHO) has supplied new guidelines to target elimination of schistosomiasis by interrupting transmission^30^. Intrinsic factors such as age and their related exposure have a possible importance in interrupting transmission when moving towards elimination^31^.

In this study, we developed a susceptible—exposed—infection—recovered—susceptible (SEIRS) dynamical model with age-related-immunity to model the transmission dynamics of *Schistosoma* infection for SAC aged 6–15 years, using published *Schistosoma* age-stratified prevalence^4^ from a study population considering school-children in 12 schools, and proximity to snail-infested water as a proxy for exposure to the *Schistosoma*. We fit the SEIRS model over prevalence data via maximum likelihood to improve our understanding of *Schistosoma* transmission dynamics and what drives SAC age-profiles. The main aims of the study were the following: (i) To identify the main determinants of *Schistosoma* infection risk, (ii) To determine whether school distance from the lake shoreline determines the exposure of SAC *Schistosoma* risk and (iii) To improve our understanding of the non-linear relationship between age and prevalence found by Reed et al*.*^4^, and whether this was partially due to the exposure to the snails or immunity developed by the SAC. Hence, we produce models with immunity to try to reproduce the age-profiles of these SAC found in the previous study^4^. Consequently, we hope this analysis will help improve understanding of *Schistosoma* transmission dynamics and in turn, improve the application of schistosomiasis control within SAC.

## Methods

We describe our modelling approach, We provide a brief description of our schistosomiasis prevalence training dataset, the state transition model used to model the disease process in the school, and how this relates to the observation process. All data processing and analysis was performed in R version 4.1.1. All methods were performed in accordance with the relevant guidelines and regulations.

### Dataset

This secondary analysis study uses the outcomes of our previously published, Reed et al*.*^4^ on *Schistosoma* age prevalence profiles and Reed et al*.*^32^ on geospatial modelling of snail distributions based on the parasitological and malacological data from Kayuni et al*.*^8^ and Al-Harbi et al*.*^33^. These data were collected in 2019 for the age-profiles and the snail distribution predictions were interpolated from aggregated data from 2017 to 2019 along the southern part of the Lake Malawi shoreline. Therefore, in the study we use cross sectional single point in time data.

We removed St Augustine 2 school from *Biomphalaria* sp. dataset as it had no variation in prevalence (effectively 1). For *Bulinus* spp. all schools in the dataset were used including St Augustine 2.

### Disease model formulation

**Fig. 1:**
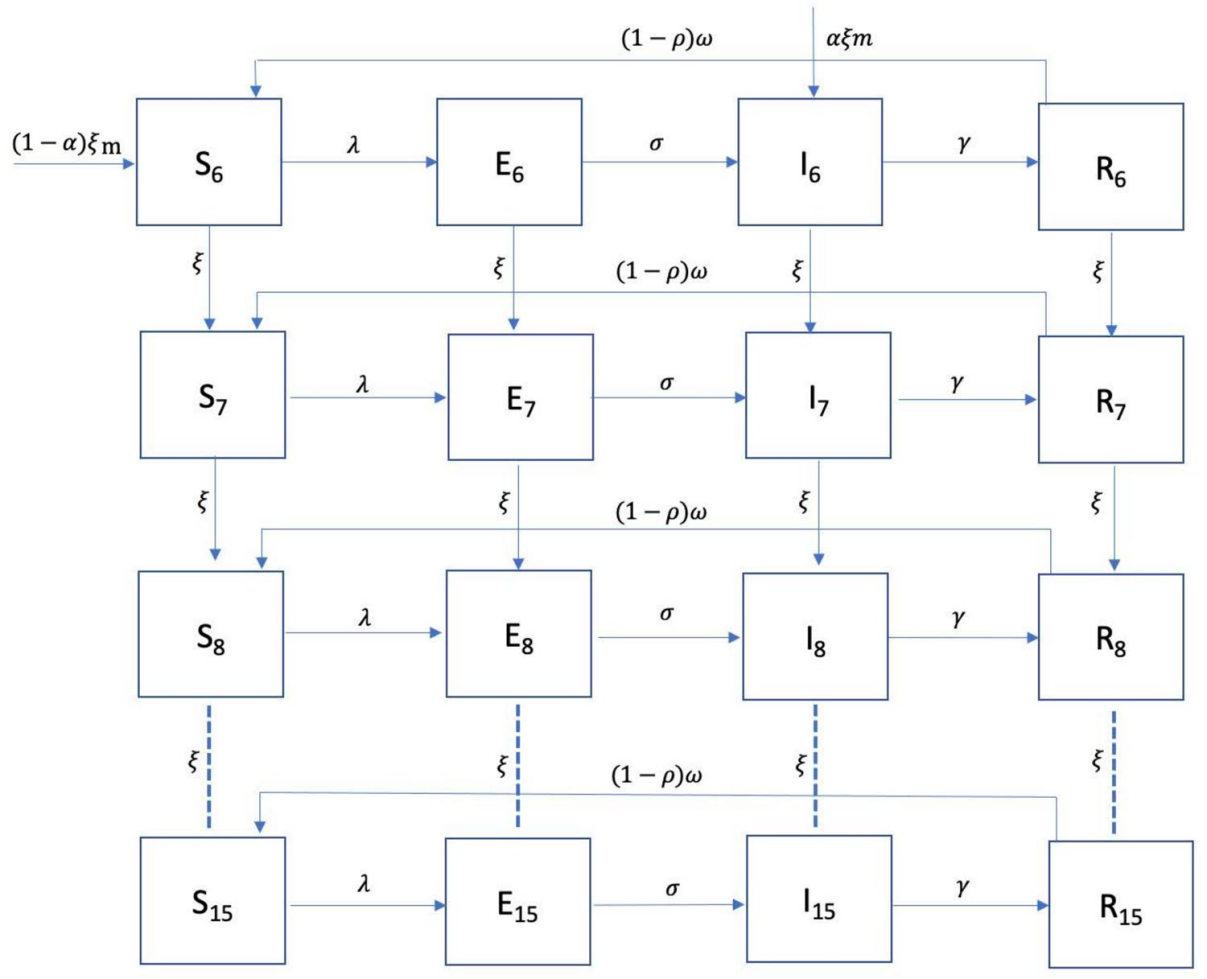
SEIRS transmission compartment model with SAC age ranging from 6 to 15 years. Transmission parameters are discussed in the text.

We consider the incidence of schistosomiasis in SAC within a school as a function of childrens’ exposures to snail-infested waters. To investigate how their age and proximity to snail habitats affects the age-prevalence relationship in SAC, we develop an age-stratified SEIRS model where children are assumed to start susceptible (S) to infection, progress to being exposed (E) (i.e. infected but not yet infectious), infectious (and detectable, I), and recovered (R) with immunity as shown in Fig. 1. We then allow for immunity to wane and the children to return to being susceptible. Our approach differs from other published models in that we do not attempt to model the dynamics of schistosomiasis in the entire populations^34,35^. Given that our sampling frame represents a very small fraction of the total population in the study region, we concentrate on modelling *Schistosoma* challenge from exposure to snails, assuming that our infected SAC have negligible effect on *Schistosoma* prevalence in the snail population.

Within each school we assume that children are divided into 10 age-grades, with a class of size of 30, represented as $m$. Children are assumed to enter school at age 6 years at a rate of $\frac{1}{30}$ per year (i.e. 30 children entering the school per year) having had no prior infection by *Schistosoma* spp. ($\alpha =0\%)$ infection. We also test the assumption where prior *Schistosoma* infection for SAC entering into age group 6 (age 6 years), $\alpha$ by testing different values (see 2.5 and 3.5 sensitivity analysis). They then progress between the age classes at a rate of $\frac{1}{365}$per day giving $\upxi$ as an aging rate of the SAC. We assumed SAC only enter the school via age 6 years and do not leave the school till aged 15 years.

Given the states, we model the rate of transition between S and E according to a function of exposure to snail habitats, mediated by distance to the lake shore. Letting ${\lambda }_{i}$ be the infection rate experienced by a child in location i, we have:

1 $$\lambda _{i} = \int \beta K_{{ij}} a\left( {x_{j} } \right){\mathrm{d}}x_{j} \simeq \frac{\beta }{n}\sum\limits_{{j \in {\mathrm{x}}}} {K_{{ij}} a\left( {x_{j} } \right),}$$

where, β (days^−1^) is the transmission rate coefficient for *Schistosoma* infection, ${K}_{ij}={\mathrm{exp}}^{(-\frac{\parallel {x}_{i}^{2}-{x}_{j}^{2}{\parallel }^{2}}{{\phi }^{2}})}$ is a function that decays with Euclidean distance between i and j, $a\left({x}_{j}\right)$ is the snail abundance (count of snails), ${x}_{j}$ is the location of snails (see Supplementary Fig. S1 online) and ${x}_{i}$ is the location of the school for each SAC, $i$ for $i=1,\cdots ,n.$ Contact with free living cercariae was assumed to be the only means through which children become infected (i.e. transition from S to E). Furthermore, all snails were assumed infected and snails’ infection status is assumed independent of contact with SAC for the reason outlined above. In addition, we assume that SAC do not infect each other, and no mother-to-child transmission of disease occurs among humans. These assumptions are supported by the fact that we only model school-children, who are themselves a small fraction of the wider population and can therefore be assumed to be approximately independent within the overall human-snail-schistosome lifecycle.

Then we model the rate of transition from E to I as σ (days^−1^), which is the rate at which immature worms become adult worms residing in intestinal or urogenital region and producing eggs. Then we model the rate of transition from I to R, with γ (days^−1^) as the rate of infective SAC moving to the R. Next, we assume that the rate of age-specific loss of immunity, $(1-\rho \left(age\right))\omega$ i.e. age-related, such that the rate of transition from R to S (Fig. 1) follows a logistic growth rate with an inverse relation to age represented as:

2 $$\rho \left(age\right)=\frac{1}{1+{e}^{-\kappa \left(age-C\right)}}$$

where $\kappa$ governs the rate of change with respect to age, and C is a constant which controls the midpoint i.e. the age at which $\rho \left(age\right)=\frac{1}{2}$ per day. For parameter identifiability reasons, we assume C to be 11 years which is the midpoint between age groups 6–15 years and was the age of peak infection prevalence found in the published secondary analysis paper, Reed et al.^4^ on *Schistosoma* prevalence age-profiles. We assumed the older the child, the slower they transition from R to S. In any transition of the model, no deaths from schistosomiasis or MDA treatment were considered.

The age-structured dynamics of schistosome transmission in this system are then represented by the following differential equations:

3 $$\frac{\overrightarrow{d{S}_{t}}}{dt}=-\left(\overrightarrow{\lambda }+\upxi \right)\odot \overrightarrow{{S}_{t}}+\upomega \left(1-\overrightarrow{\rho }\right)\odot \overrightarrow{{R}_{t}}+\upxi \overrightarrow{{S}_{t}^{+}}$$

4 $$\frac{\overrightarrow{d{E}_{t}}}{dt}=\overrightarrow{\lambda }\odot \overrightarrow{{S}_{t}}-\left(\upsigma +\upxi \right)\overrightarrow{{E}_{t}}+\upxi \overrightarrow{{E}_{t}^{+}}$$

5 $$\frac{\overrightarrow{d{I}_{t}}}{dt}=\upsigma \overrightarrow{{E}_{t}}-\left(\upgamma +\upxi \right)\overrightarrow{{I}_{t}}+\upxi \overrightarrow{{I}_{t}^{+}}$$

6 $$\frac{\overrightarrow{d{R}_{t}}}{dt}=\upgamma \overrightarrow{{I}_{t}}-\left(\upxi +\upomega \left(1-\overrightarrow{\rho }\right)\right)\odot \overrightarrow{{R}_{t}}+\upxi \overrightarrow{{R}_{t}^{+}}$$

where, *t* stands for time in days.

The age structure of the dynamical model are represented as the following equations:

7 $$\left\{\overrightarrow{{S}_{t}^{+}}\right\}={((1-\alpha )m, {S}_{t}^{6},{S}_{t}^{7},{S}_{t}^{8},{S}_{t}^{9},{S}_{t}^{10},{S}_{t}^{11},{S}_{t}^{12},{S}_{t}^{13},{S}_{t}^{14})}^{T},$$

8 $$\left\{\overrightarrow{{E}_{t}^{+}}\right\}={(0, {E}_{t}^{6},{E}_{t}^{7},{E}_{t}^{8},{E}_{t}^{9},{E}_{t}^{10},{E}_{t}^{11},{E}_{t}^{12},{E}_{t}^{13},{E}_{t}^{14})}^{T},$$

9 $$\left\{\overrightarrow{{I}_{t}^{+}}\right\}={(\alpha m, {I}_{t}^{6},{I}_{t}^{7},{I}_{t}^{8},{I}_{t}^{9},{I}_{t}^{10},{I}_{t}^{11},{I}_{t}^{12},{I}_{t}^{13},{I}_{t}^{14})}^{T},$$

10 $$\left\{\overrightarrow{{R}_{t}^{+}}\right\}={(0, {R}_{t}^{6},{R}_{t}^{7},{R}_{t}^{8},{R}_{t}^{9},{R}_{t}^{10},{R}_{t}^{11},{R}_{t}^{12},{R}_{t}^{13},{R}_{t}^{14})}^{T}.$$

For a given set of parameters, $\theta =(\beta ,\sigma ,\gamma )$, we solve this set of ODEs using Euler’s method as implemented in the R package “desolve” version 4.1.1 (reduce computation time required). The solver is run until the SEIRS system achieves equilibrium, which by experimentation we find to be by timestep $t=1000$ days for a large range of parameter values.

For simplicity, and exposition of the inference methods in the next section, we abstract the ODE model into a mathematical function, of the parameters $\theta$ and initial conditions ${X}_{0}=\left\{\overrightarrow{{S}_{0}},\overrightarrow{{E}_{0}},\overrightarrow{{I}_{0}},\overrightarrow{{R}_{0}}\right\}$.

11 $${S}^{*},{E}^{*},{I}^{*},{R}^{*}=g(\theta ,{x}_{0,} z),$$

where the vector ${S}^{*},{E}^{*},{I}^{*},{R}^{*}$ denotes the number of children in each age-group in each epidemiological state at equilibrium, $z$ represents our input data (snail abundance, distance to the shoreline), and $\theta =(\beta ,\sigma ,\gamma )$ our model parameters. Where necessary, we subscript these quantities to denote that they relate to a specific school, for example ${\theta }_{s}=({\beta }_{s},\sigma ,\gamma )$ to denote the condition where all schools share $\sigma$ and $\gamma$ but have individual ${\beta }_{s}$ for the $s$ th school.

### Observation of prevalence

From our study we have observation of childhood infection prevalence in school. For school $s$, given that ${S}_{s}^{*},{E}_{s}^{*},{I}_{s}^{*},{R}_{s}^{*}=g(\theta ,{x}_{0}, z)$, we assume observed number of positive children ${y}_{as}$

in age-group $a$ given a sample of ${n}_{a}$ children is Binomially distributed given that.

12 $${y}_{as}\sim Binomial({n}_{as},{\pi }_{as}),$$

where ${\uppi }_{\mathrm{as}}={\mathrm{I}}_{\mathrm{as}}^{*}/{\mathrm{N}}_{\mathrm{as}}$ where ${\mathrm{I}}_{\mathrm{as}}^{*}$ is the modelled number of infected children in age-group $a$ in school $s$, and ${N}_{as}=30$ is the size of the class.

### Inference

In the model described above, we have parameters $\beta ,\sigma ,\gamma$ which remain unknown. In order to estimate these we fitted the model using maximum likelihood estimation. Since the ODE model is a deterministic function of the input parameters and covariate data, the log-likelihood is a product over the 10 age groups and 12 schools.

13 $$l\left(\theta ; \pi ,n\right)\propto \sum_{a=1}^{10}\sum_{s=1}^{12}{y}_{as }\mathrm{log}({\pi }_{as})+({n}_{as}-{y}_{as})\mathrm{log}(1-{\pi }_{as}) + k,$$

where *k* is a constant. We calculated estimates for $\theta$ by optimising the log-likelihood numerically using the L-BFGS-B method provided by R’s “optim” function. Parameters bounded at zero were log-transformed for ease of optimisation, with marginal log-likelihoods generated for each parameter to visually inspect the quality of optimisation and suggest starting points for the optimisation algorithm. Parallel computing was used to quicken the computer processing time, exploiting conditional independence of the schools in the model, enabling the ODE solvers for each school to run simultaneously.

**Fig. 2:**
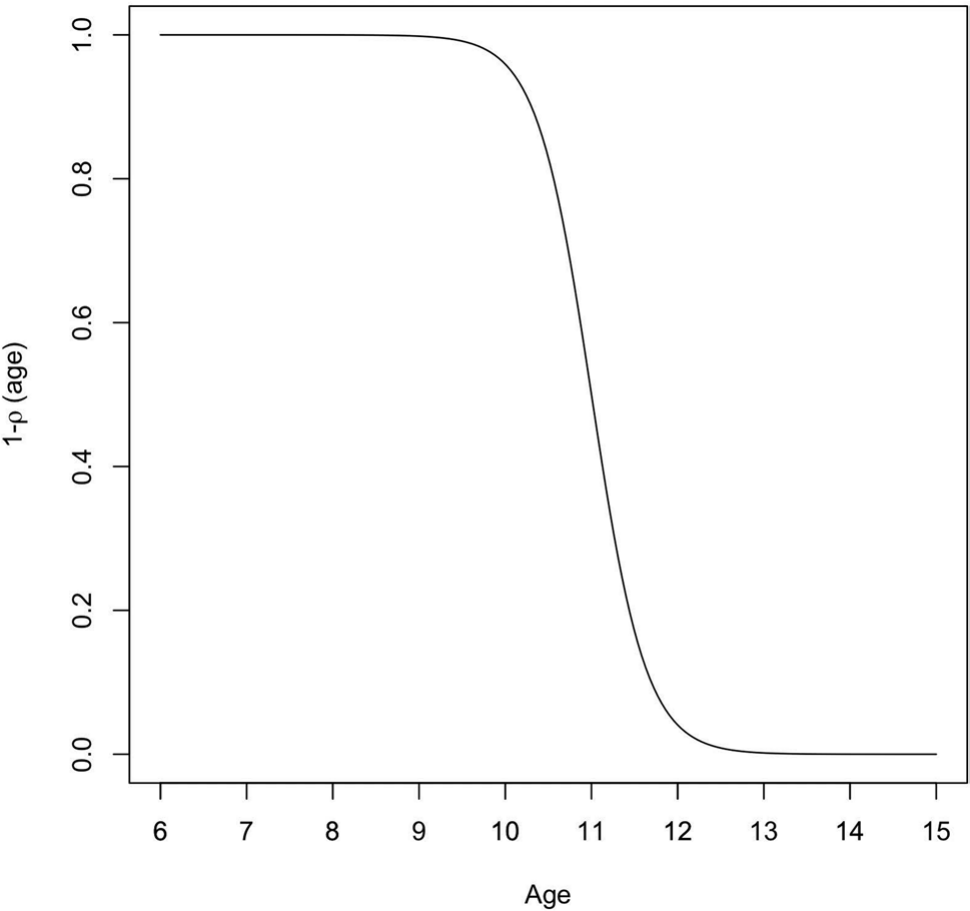
Logistic age-specific loss of immunity function, $1-\rho \left(age\right)$ versus age. For $\kappa =0.5$ days^−1^, $C=11$ years, age ranging between 6 and 15 years.

Due to data in this model being cross-sectional data for a single point in time, we cannot easily identify a timescale for the disease process. If we double all transition rates, we find the same steady state conditions. To accommodate for this, we chose to fix $\kappa$ (smoothness of age - dependent) and $\omega$ (recovery of loss of immunity of SAC aged 6 years) for identifiability purposes. Since $\beta ,\gamma ,\sigma$ are rate parameters (days^−1^), and therefore have positive support, they were optimised on the log scale, e.g. optimising ${\beta }^{*}=\mathrm{log}\left(\beta \right).$ This improved the efficiency of the optimisers by constraining the parameter space to be positive. $\kappa$ was fixed to be $\kappa =0.5$ days^−1^ as this allowed for a moderate age-related loss of immunity slope in the logistic regression function with $C=11.0$ years as shown in Fig. 2. $\omega$ was fixed till $\upomega =0.5$ days^−1^ such that the rate of loss of immunity was approximately 0.5 days^−1^ for 6-year-olds. We also fixed the spatial decay parameter $\phi$ which enabled the identification of the $\beta$ parameters given the length scale. $\phi$ was fixed at $\phi =4.48$km as represents spatial decay to 0 by 100 km (the approx. distance along the shoreline of Lake Malawi for our data used) and the distance was divided by 10km to improve the numerical stability and convergence of the model and avoid overfitting reducing the computational complexity.

To allow the optimiser to find the best-estimate values of the parameters in our models with reduced computation time, we set initial values for the non-fixed parameters by plotting the marginal log-likelihoods and starting the optimiser near to the maximum (peak) of the log-likelihood. We constrained the optimiser search space by $\pm 1$ either side of the graphical maximum to stop the optimiser entering unstable regions of the parameter space^36^.

The log-likelihood was plotted against each parameter (keeping the other parameters fixed at their respective MLE) to check if the maximum was found. Optimisation of certain parameters was carried out as shown in Table 1.

**Table 1:** Different models used in the optimiser to find the best estimated parameters values.

Parameters	Definition	Multi-${\beta }_{s}$ space	Single-$\beta$ space	Multi-${\beta }_{s}$ no space	Single-$\beta$ no space
$\upbeta$	Transmission rate coefficient (days^−1^)	Multiple	Single	Multiple	Single
$\upsigma$	Rate of exposed SAC becoming infective (shredding eggs) (days^−1^)	Unknown	Unknown	Unknown	Unknown
$\upgamma$	Rate of infective SAC Recovering (days^−1^)	Unknown	Unknown	Unknown	Unknown
$\upxi$	School recruitment rate and rate at which SAC age	$\frac{1}{365}$ days^−1^			
$\kappa$	Smoothness of age-dependent loss of immunity curve (logistic curve)	0.5 days^−1^			
C	Constant (Age range of SAC)	11.0 years			
$\upomega$	Recovery of loss of immunity of SAC aged 6 years	0.5 days^−1^			
$\phi$	Spatial decay constant	4.48km	4.48km	${10}^{6}$km	${10}^{6}$km

The following four models were fitted for each species using the optimiser as shown in Table 1.

1. Different (independent) ${\beta }_{s}$ value (transmission rate) for each school $s$ with spatial effect
2. single-$\beta$ value for all schools with spatial effect
3. No spatial effect by making $\phi$ large with multiple (independent) ${\beta }_{s}$’s values for each school
4. No spatial effect by making $\phi$ large with single-$\beta$ value for all schools.

In the third and fourth model we removed the spatial effect competent, modifying the snail force of infection to test whether we still need a model with multi-${\beta }_{s}$’s or single-$\beta$ for each school.

We then calculated the Akaike Information criterion (AIC) to compare the fit of different models, and is our preferred metric as it trades off increasing fit to the data with model complexity. In additional, we examined the in-sample predictive performance of the model through visual assessment of predicted versus observed prevalence values. In addition, to assess the in-sample predictive performance further, mean squared error (MSE) was computed for each model for each school to compare between the observed and predicted prevalence using the following:

14 $$\frac{1}{n}\sum\limits_{{i = 1}}^{n} {(Y_{i} - \widehat{{Y_{i} }})^{2} }$$

where a vector of $n$ predictions were sampled from our $n$ data points ($i=1,\dots ,n)$ for all variables, ${Y}_{i}$ is the vector of the observed values of prevalence (at school location ${x}_{i}$) that are being used for the prediction and $\widehat{{Y}_{i}}$ are the predicted parameter values of model. Then we took the mean of Eq. (14) (e.g. square of the errors) to find MSE value for each model.

We computed approximate confidence intervals (CI) for our parameter estimate using the Wald method based on the diagonal of the Fisher Information matrix. Then we plotted the estimated coefficients values (parameters) and the CI together.

### Sensitivity analysis

A sensitivity analysis was carried out by allowing for prior *Schistosoma* infection prevalence of SAC entering age group 6, $\alpha$ as 5% (0.05), 10% (0.10) and 20% (0.20).

## Results

### Check best value of ${\boldsymbol{\phi}}$

If we fixed all parameters to the estimated values from multi-${\beta }_{s}$ space model and unfix $\phi$ the model converges quickly and finds the best value of $\phi$=4.48km.

#### Snail abundance

**Fig. 3:**
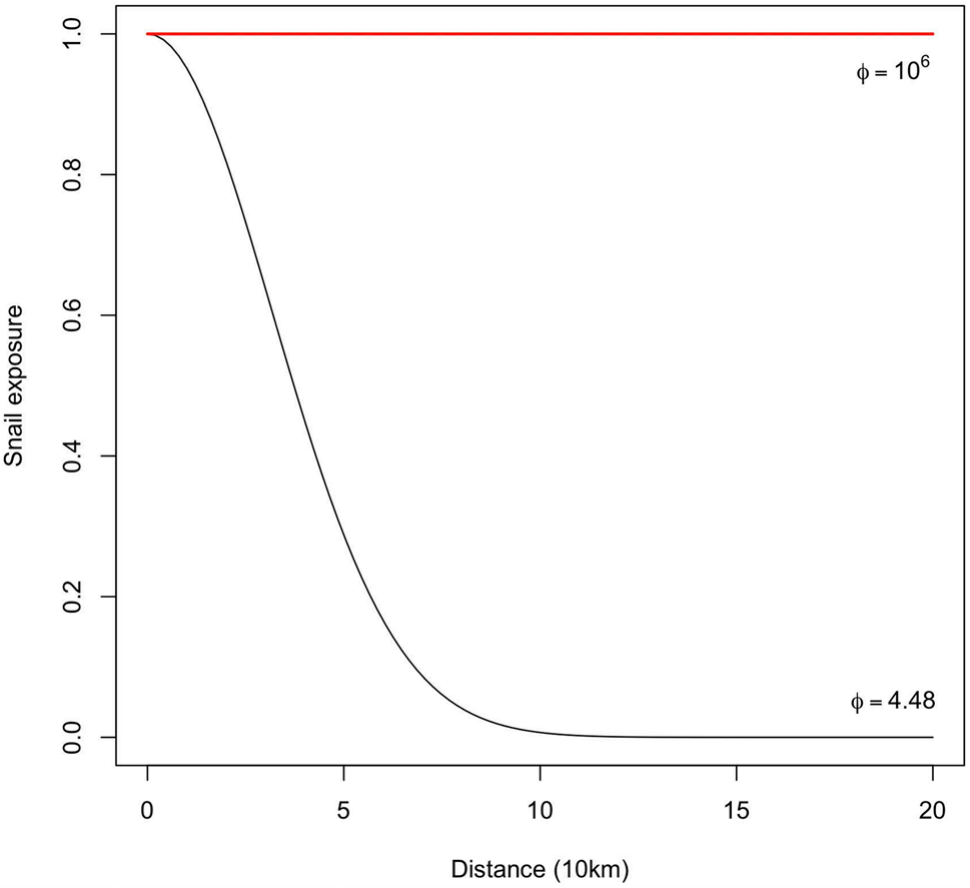
Distance (10km) of school from lake shoreline versus SAC snail-infested water exposure . Black line: $\phi$ = 4.48km, red line: $\phi ={10}^{6}$km.

Figure 3 shows the distance (10km) of school from the lake shoreline versus SAC snail-infested water exposure decreases till $\sim$100km distance from school to shoreline when $\phi$=4.48km. Whereas, when $\phi ={10}^{6}$ km, the snail exposure is the same all along the shoreline, which shows there is no spatial effect in this case.

### Model optimisation

All models converged to the maximum likelihood for each school (See Supplementary Figs. S2–S7 online) although there were some identifiability issues between $\beta$ and $\gamma$. As shown in Tables 2, 3, 4 and 5 the following was found: the best model (AIC = 250) for *Biomphalaria* sp. was the single-$\beta$ with no spatial effect. When testing multi-${\beta }_{s}$ or single-$\beta$ with spatial effect (AIC = 258 & AIC = 269) and multi-${\beta }_{s}$ with no spatial effect (AIC = 258) we found a worse model fit compared to single-$\beta$ with no spatial effect (AIC = 250). Whereas for *Bulinus* sp. the best model (lowest AIC) was the multi-${\beta }_{s}$ with no spatial effect (AIC = 248). When testing the multi-${\beta }_{s}$ with spatial (AIC = 250), single-$\beta$ with spatial effect (AIC = 341) and single-$\beta$ with no spatial effect (AIC = 351) we found a worse model fit compared to the multi-${\beta }_{s}$ with spatial effect (AIC = 248).

**Table 2:** Parameter estimates for multi-${\beta }_{s}$ spatial model for each species.

Multi-${\beta }_{s}$space								
	*Biomphalaria* AIC = 258			MSE	*Bulinus* AIC = 250			MSE
Parmeters/Schools [CI]	$\mathrm{log}\: \beta$	$\mathrm{log}\:\sigma$	$\mathrm{log}\: \gamma$		$\mathrm{log}\: \beta$	$\mathrm{log}\: \sigma$	$\mathrm{log}\: \gamma$	
Mchoka	− 2.78 [− 5.31, − 0.253]	− 2.59 [− 9.07, 3.89]	− 5.40 [− 7.68, − 3.13]	0.442	− 6.31 [− 8.30, − 4.31]	− 2.80 [− 7.93, 2.33]	− 4.65 [− 6.50, 2.80]	0.0661
Samama	− 2.15 [− 5.83, 1.54]			0.339	− 4.15 [− 7.42, − 1.62]			0.254
MOET	− 4.65 [− 8.45, − 0.85]			0.252	− 7.32 [− 9.38, − 5.25]			0.0656
Koche	− 2.97 [− 13.1, 7.15]			0.103	− 8.95 [− 11.65, − 6.26			0.0248
St Augustine 2					− 5.02 [− 7.45, − 2.59]			1.15
Ndembo	− 0.922 [− 4.49, 2.64]			0.186	− 4.38 [− 7.58, − 1.18]			0.657
Sungusya	1.66 [− 7.96, 11.3]			0.380	− 6.24 [− 8.38, − 4.09]			1.18
St Martins	1.72 [− 9.70, 13.1]			0.420	− 8.26 [− 11.0, − 5.56]			0.0564
Chikomwe	− 1.77 [− 5.28, 1.74]			0.487	− 7.54 [− 9.76, − 5.33]			0.261
Chipeleka	0.769 [− 8.82, 10.4]			0.291	− 6.14 [− 8.30, − 3.96]			0.397
Makumba	0.438 [− 2.74, 3.61]			0.671	[− 9.8,− 5.10]			0.254
Mtengeza	0.919 [− 3.52, 5.35]			0.451	5.84 [− 8.04 ,− 3.64]			0.870

**Table 3:** Parameter estimates for single-$\upbeta$ spatial model for each species.

Single-$\upbeta$ space								
	*Biomphalaria* AIC = 269			MSE	*Bulinus* AIC = 341			MSE
Parmeters/Schools [CI]	$\mathrm{log}\:\beta$	$\mathrm{log}\:\sigma$	$\mathrm{log}\:\gamma$		$\mathrm{log} \beta$	$\mathrm{log}\:\sigma$	$\mathrm{log}\:\gamma$	
Mchoka	− 0.514 [− 0.526, − 0.502]	− 4.60 [− 5.18, − 4.02]	− 6.46 [− 7.00, − 5.92]	0.684	− 5.00 [− 9.07, − 0.927]	0.500 [− 12.2, 13.2]	− 3.55 [− 7.58, 0.489]	0.0856
Samama				0.357				1.17
MOET				0.291				0.307
Koche				0.107				0.459
St Augustine 2								1.49
Ndembo				0.134				1.64
Sungusya				0.469				1.12
St Martins				0.681				0.482
Chikomwe				0.475				0.859
Chipeleka				0.307				0.400
Makumba				0.894				0.455
Mtengeza				0.756				0.909

**Table 4:** Parameter estimates for multi-${\upbeta }_{\mathrm{s}}$ no spatial model for each species.

Multi-${\upbeta }_{\mathrm{s}}$ no space								
	*Biomphalaria* AIC = 258			MSE	*Bulinus* AIC = 248			MSE
Parameters/Schools [CI]	$\mathrm{log}\:\beta$	$\mathrm{log}\:\sigma$	$\mathrm{log}\:\gamma$		$\mathrm{log}\:\beta$	$\mathrm{log}\:\sigma$	$\mathrm{log}\:\gamma$	
Mchoka	− 6.07 [− 8.59, 3.56]	− 2.59 [− 9.08, 3.90]	− 5.40 [− 7.66, − 3.15]	0.442	− 7.26 [− 12.4, − 2.14]	− 2.50 [− 8.49, 3.38]	− 3.39 [− 8.49, 1.72]	0.0683
Samama	− 5.12 [− 8.79, − 1.45]			0.339	− 4.80 [− 11.0, 1.35]			0.215
MOET	− 5.07 [− 8.86, − 1.29]			0.252	− 8.26 [− 13.4, − 3.12]			0.0682
Koche	− 3.80 [− 13.9, 6.29]			0.103	− 9.98 [− 15.4, − 4.55]			0.0249
St Augustine 2					− 5.73 [− 11.2 ,− 0.289]			1.08
Ndembo	− 5.23 [− 8.78, − 1.68]			0.186	− 4.20 [− 12.1, 3.70]			0.623
Sungusya	− 3.89 [− 13.5, 5.68]			0.380	− 7.41 [− 12.6, − 2.19]			1.13
St Martins	− 3.73 [− 15.1, 7.66]			0.420	− 9.26 [− 14.7, − 3.81]			0.0568
Chikomwe	− 5.26 [− 8.75, − 1.76]			0.486	− 8.05 [− 13.3, − 2.82]			0.261
Chipeleka	− 3.90 [− 13.4, 5.63]			0.291	− 6.72 [− 12.0, − 1.50]			0.390
Makumba	− 5.50 [− 8.66, − 2.34]			0.672	− 8.51 [− 13.8, − 3.22]			0.256
Mtengeza	− 4.91 [− 9.33, − 0.489]			0.451	− 6.53 [− 11.8, − 1.24]			0.884

**Table 5:** Parameter estimates for single-$\upbeta$ no space model outcome for each species.

Single-$\upbeta$ no space								
	*Biomphalaria* AIC = 250			MSE	*Bulinus* AIC = 351			MSE
*Parmeters*/*Schools [CI]*	$\mathrm{log}\:\beta$	$\mathrm{log}\:\sigma$	$\mathrm{log}\:\gamma$		$\mathrm{log}\:\beta$	$\mathrm{log}\:\sigma$	$\mathrm{log}\:\gamma$	
Mchoka	− 1.48 [ − 4.95, 2.00]	0.147 [− 3.34, 3.63]	− 2.61 [ − 2.85, − 2.36	0.613	− 5.50 [− 14.8, 3.75]	− 4.62 [− 11.8, 2.64]	− 3.70 [8.67, 1.27]	0.100
Samama				0.275				1.24
MOET				0.190				0.329
Koche				0.233				0.547
St Augustine 2								1.45
Ndembo				0.261				1.78
Sungusya				0.519				1.16
St Martins				0.372				0.541
Chikomwe				0.535				0.542
Chipeleka				0.476				0.390
Makumba				0.506				0.541
Mtengeza				0.326				0.924

MSE score was the same for *Biomphalaria* sp. multi-${\beta }_{s}$ with spatial effect and no spatial effect. For both *Biomphalaria* sp. and *Bulinus* spp., there were cases where the MSE was slightly lower (better value) for particular parameters estimates for a school despite the model having a higher AIC score compared to the other models. For instance, *Biomphalaria* sp. multi-${\beta }_{s}$ with spatial effect (AIC = 258), Chikomwe school MSE score was 0.487 whereas for single-$\beta$ with spatial effect (AIC = 269) Chikomwe school MSE score was 0.475.

From the best fit model, for *Biomphalaria* sp. the following parameter values were found for all schools, $\mathrm{log}\:\beta$ [− 1.42, CI − 4.95, 2.00], for $\mathrm{log}\:\sigma$ [0.147, CI − 3.34, 3.63] giving $\frac{1}{\sigma}=0.863$ days [ranging from 0.0265 to 28.2 days] exposed period and for $\mathrm{log}\:\gamma$ [− 2.61, CI − 2.85, − 2.36] giving $\frac{1}{\gamma }=13.6$ days [ranging from 0.0573 to 17.3 days]. For the best fit model, for *Bulinus* spp. the following parameter values were found all schools, $\mathrm{log}\:\sigma$ was estimated to be − 2.50 [CI − 8.49, 3.38] giving $\frac{1}{\sigma}=12.2$ days [ranging from 0.03 to 4870 days] and $\mathrm{log}\:\gamma$ [− 3.39, CI − 8.49, 1.72] giving $\frac{1}{\gamma }=29.7$ days [ranging from 0.179 to 4866 days] infectious period for all the schools. For *Bulinus* spp. at each school, the following $\mathrm{log}\:\beta$ parameters were found: Mchoka [− 7.26, CI 12.4, − 2.14], Samama [− 4.80, CI − 11.0, 1.35], MOET [-8.26, CI − 13.4, − 3.12], Koche [− 9.98, CI − 15.4, − 4.55], St Augustine 2 [− 5.73 CI − 11.2,0. 289], Ndembo [− 4.20, CI − 12.1, 3.70], Sungusya [− 7.41, CI − 12.6,-2.19], St Martins [− 9.26, CI − 14.7, − 3.81], Chikomwe [− 8.05, CI − 13.3, − 2.82], Chipeleka [− 6.72, CI − 12.0, − 1.50], Makumba [− 8.51, CI − 13.8, − 3.22], Mtengeza [− 6.53, CI − 11.80, − 1.24].

**Fig. 4:**
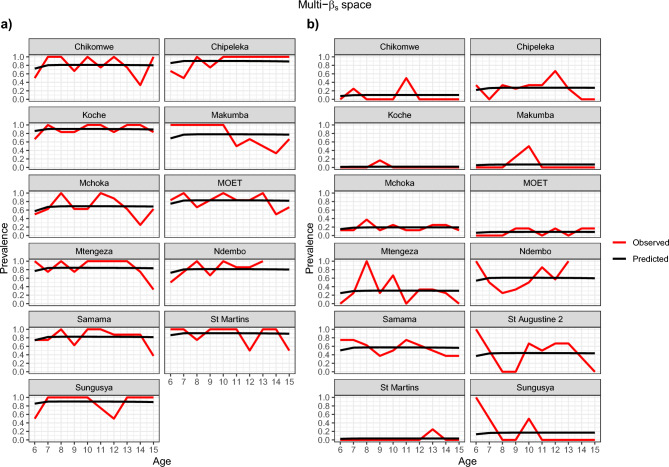
Multi-β_s_ with space effect model optimisation prevalence prediction (black line) and observed prevalence (red line) against age of SAC carried out for each species. (**a**) *Biomphalaria* sp., (**b**) *Bulinus* spp.

**Fig. 5:**
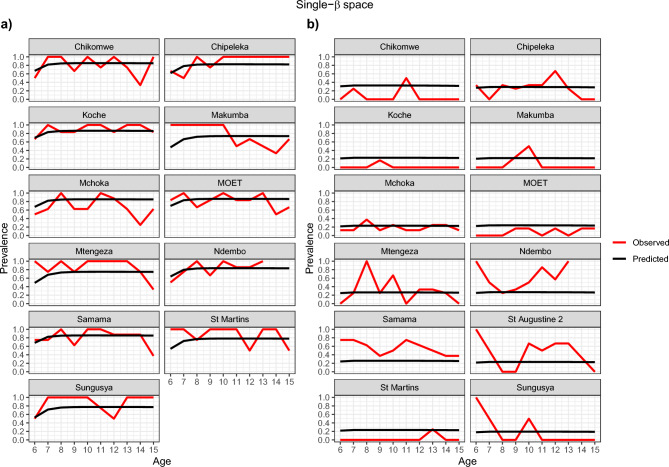
Single-β with space effect model optimisation prevalence prediction (black line) and observed prevalence ( red line) against age of SAC carried out for each species. (**a**) *Biomphalaria* sp., (**b**) *Bulinus* spp.

**Fig. 6:**
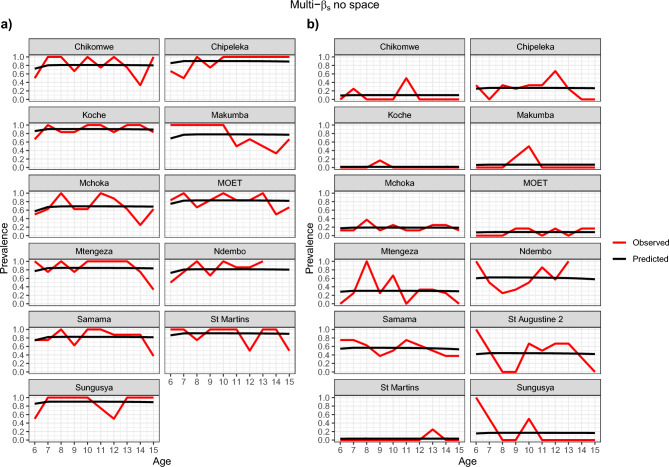
Multi-β_s_ with no space effect model optimisation prevalence prediction (black line) and observed prevalence (red line) against age of SAC carried out for each species. (**a**) *Biomphalaria* sp., (**b**) *Bulinus* spp.

**Fig. 7:**
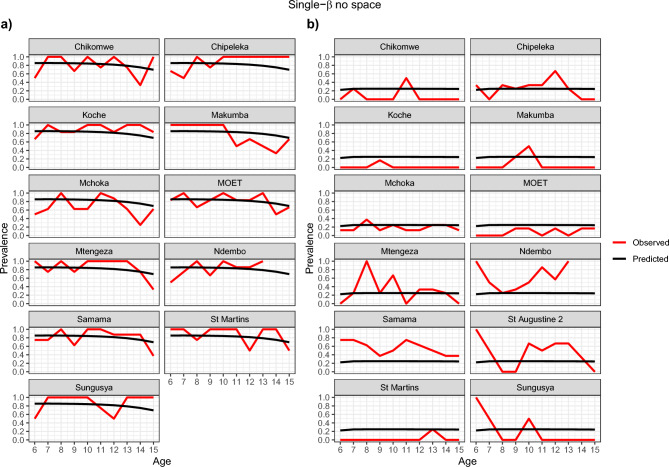
Single-β with no space effect model optimisation prevalence prediction, ${\widehat{\pi }}_{as}$ (black line) and observed prevalence (red line) against age of SAC carried out for each species. (**a**) *Biomphalaria* sp., (**b**) *Bulinus* spp.

Figures 4, 5, 6 and 7 show visually that the prevalence prediction model, ${\widehat{\pi }}_{as}$ at steady state was able to capture the age-prevalence structure of the Reed et al. 2023 paper^4^. Visually there is no evidence to suggest these models do not fit the data for *Biomphalaria* sp. with either single-$\beta$ or multi-${\beta }_{s}$ cases and for *Bulinus* spp. only the multi-${\beta }_{s}$ case. For *Bulinus* spp. the single-$\beta$ does not visually capture the observed prevalence model for certain schools including Samama, Moet, Koche, Ndembo and St Martins.

### CI intervals

**Fig. 8:**
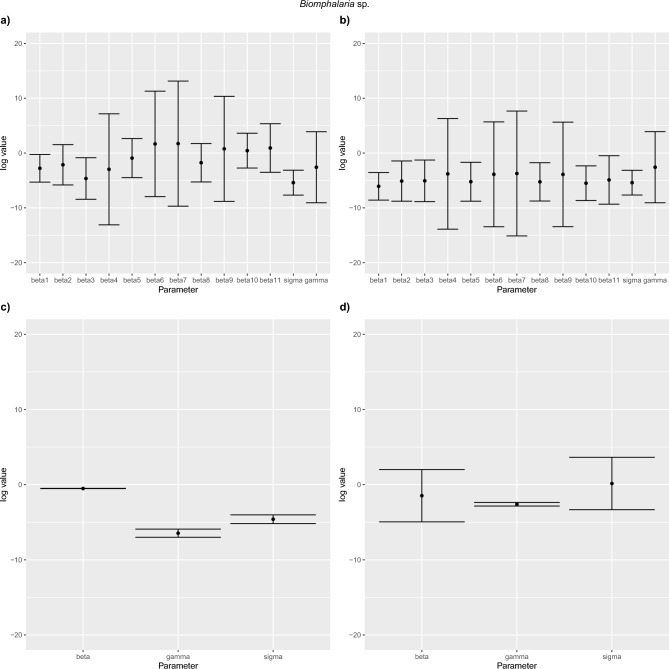
Confidence intervals for parameter estimates for *Biomphalaria* sp. models with SAC prevalence at age 6 set as α set to zero. (**a**) Multi-β_s_ space, *Biomphalaria* sp., (**b**) Multi-β_s_ no space, *Biomphalaria* sp., (**c**) Single-β space, (**d**) Single-β no space.

**Fig. 9:**
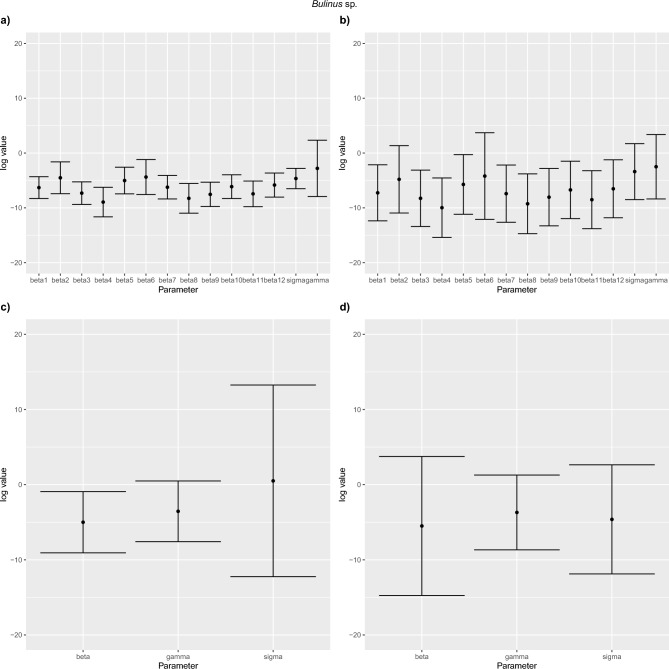
Confidence intervals for parameter estimates for *Bulinus* spp. models with SAC prevalence at age 6 set as α set to zero (**a**) Multi-β_s_ space, (**b**) Multi-β_s_ no space, (**c**) Single-β space, (**d**) Single-β no space.

We assessed the likely impact of school-level differences in baseline transmission rate by comparing $\mathrm{log}\:\beta$ values. Considering $\mathrm{log}\:{\widehat{\beta }}_{1}$ as a reference, the confidence intervals for all other $\mathrm{log}\:{\widehat{\beta }}_{2, \dots ,12}$ include $\mathrm{log}\:{\widehat{\beta }}_{1}$ such that we have no strong evidence that schools 2,…, 12 differ from school 1. In other words, a single ${\beta }_{1}$ for all schools (single-$\beta$ model) in the model suffices (Figs. 8 and 9). For *Biomphalaria* sp. multi-${\beta }_{s}$ space and no space models, Fig. 8a and b shows visually the approximate baseline ${\beta }_{1}$ value to be between $\mathrm{log}\:\beta =-3$ and $\mathrm{log}\:\beta =-7$ from average of the CIs for all schools. *For Biomphalaria* sp. single-$\beta$ space and no space models, Fig. 8c and d shows visually the approximate baseline ${\beta }_{1}$ value to be between $\mathrm{log}\:\beta =-0.5$ and $\mathrm{log}\:\beta =1.5$ from average of the CIs for all schools. For *Bulinus* spp. multi-${\beta }_{s}$ space and no space models, Fig. 9a and b shows visually the approximate baseline ${\beta }_{1}$ value to between $\mathrm{log}\:\beta =-6$ and $\mathrm{log}\:\beta = -7$ from average of the CIs for all schools. For single-$\beta$ space and no space models, Fig. 9c and d shows visually the approximate baseline ${\beta }_{1}$ value to be between $\mathrm{log}\:\beta =-5$ and $\mathrm{log}\:\beta =-5.5$ from average of the CIs for all schools.

### Sensitivity analysis

**Fig. 10:**
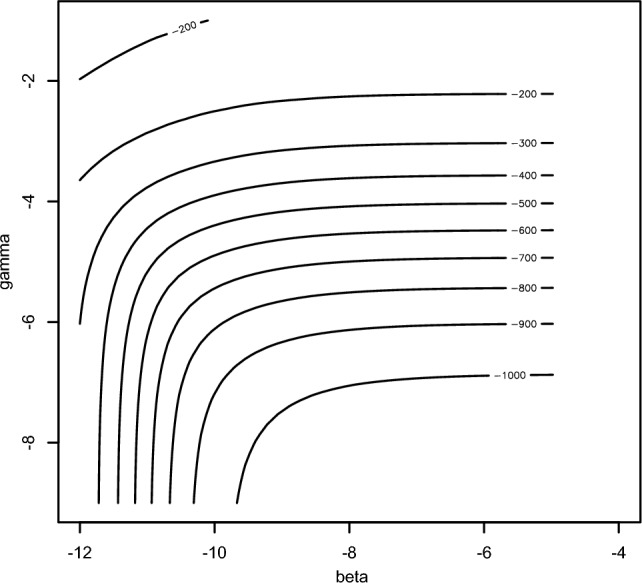
Contour plot showing non-identifiability issue between γ and β parameters.

When testing different values of SAC age 6 prevalence, $\alpha$ we found that the parameter estimates did not change a lot and the AIC and MSE scores were similar, suggesting a similar fit. In addition, for $\alpha =0.10$ and $\alpha =20$ for *Bulinus* spp. we found that that there were non-identifiability issues between $\gamma$ and $\beta$ as shown in Fig. 10 where essentially the optimiser is just contouring around the distribution, following the contours, instead of finding the estimated parameter value(s).

In addition the plots of the fits to the age-prevalence profiles for the different $\alpha$ values are shown in Supplementary Figs. S8–S19 online. The tables of the parameter estimates for the different $\alpha$ values are shown in Supplementary Tables S1–S12 online. The confidence intervals for the parameter estimates for the different $\alpha$ values are shown in Supplementary Figs. S20–S25 online.

## Discussion

Our analysis has made attempts to model the dynamics of *Schistosoma* transmission at a single point in time, and estimate the best possible values of our parameters given our data. Our model was designed to determine SAC associated *Schistosoma* infection risk and to determine whether school distance from the lake shoreline determines the exposure of SAC *Schistosoma* risk, and further, whether we can reproduce the SAC prevalence age-profiles shown in Reed et al.^4^ on our study population (i.e. school-children in 12 schools). This was conducted using a SEIRS ODE model and carried out using the LBFGS-B optimisation algorithm. Our model is novel in the sense that we focus on capturing the disease process within children as a function of exposure to a quasi-static snail population, as opposed to modelling the entire *Schistosoma* lifecycle as is common in other studies^34,35^. Our approach by comparison is more parsimonious, with the advantage of decreased complexity and increased computational speed.

Our study found the best fitting model for *Biomphalaria* sp. was the single-β no spatial effect model (AIC = 250), which supports the use of single-β model for all schools and with no spatial decay of the force of infection from snails with distance from the lake shoreline. Hence, we cannot conclude that transmission differs appreciably between schools or that school distance from the shoreline has an effect on SAC exposure. For *Bulinus* spp., however, the best model was the multi-β_s_ with no spatial effect model, which supports a hypothesis that school-level factors determine the apparent prevalence of urinary schistosomiasis though we have no evidence that the spatial relationship to snail exposure affects prevalence. Therefore, within the narrow range studied, distance from the shoreline to the schools was not a determinant.

There was possible heterogeneity in transmission between schools. Based on our AIC scores the model preferred incorporating different transmission rates for each school and this was also shown in Figs. 4 and 6 which suggests this is likely the case. In particular, one noted result was Samama, Moet, Koche, Ndembo and St Martins predictive estimate for single transmission rate for all schools with *Bulinus* spp. did not capture the data that we observed and so this suggested that we needed different transmission rates in the schools. However, we found no statistical evidence that transmission rates are different from each other based on CIs given our data. This may well be due to the noisy nature of the observed prevalences, and associated wide confidence intervals, as well as the possibility that our approximate confidence intervals were not capturing the true correlation structure in the joint likelihood surface. Since, Figs. 4, 5, 6, and 7 indicate that the multi-$\beta$ models indicate a better predictive fit, it is perhaps not surprising that these are preferred by AIC. Our conclusion, therefore, is that the multi-$\beta$ model should be preferred on the grounds of improved predictive performance, rather than model parsimony.

From the best fit models, the exposed period (S to E group) was estimated to be 0.863 days for *Biomphalaria* sp. and 12.2 days *Bulinus* spp. compared to 14–84 days [usually between 35 and 42 days] usually reported in other studies^23,37^. In additional, the infectious period (E to I group) was estimated to be 13.6 days and 0.357 days, whereas in other studies the infectious period (gamma) (I to R group) has been found to be estimated around 35 days (14–70 days); we found lower rate in the infectious period than previous studies^13^. These differences in exposed and infectious period could have been due to some identifiability issues (i.e. Fig. 10) that were noted for our unknown parameters beta, sigma, gamma making it difficult for the model to capture the observed prevalence in our dataset. Further, the shorter exposed and infectious predictions could suggest our model fit is artefact of data collection or even the ODE fitted to a small population (in which there is a continuous approximation to a discrete state space of numbers of individuals). A SIR model could have been the more parsimonious model to fit, albeit with the obvious violation of a biological principle (the fact that your infection is not immediately detectable when you first get infected).

### Limitations

A limitation of this study is that we only accounted for distance from the lake shoreline (data aligned to the lake shoreline) and did not account for inland bodies of water, for instance, ponds and pools of water inland in the dataset. Further we do not know in detail the local environment from the inland schools, only the local environment and their relative exposure along the lake shoreline. If there is any effect of differential exposure to snails by SAC at the different schools, this may not be the best representation using the simple distance from the lake. This could be due to individual human spatial movement having predictable patterns influenced by their environment and socio-economic factors. For instance, SAC schools that have direct and easy access to the lakeshore could be more likely to be exposed to snails as they visit the lake shoreline more often than rural villages^38^. This could have also been due to all our schools in our study being close enough to the lake that they guarantee SAC visiting the shoreline often. Although, water contact can also be dictated by gender or age^9^. Our study was place specific so there may be a different result at other locations with different snail species or seasonality. In the future other geospatial variables such as water quality or microhabitats within the lake could be considered. Moreover, the study could have been expanded to other schools in the area, changes to water contact patterns throughout the year and seasonality^9^.

We also consider SAC to only travel using Euclidean distance (straight) from their school to the lake shoreline. This is a misrepresentation as often SAC will go to different parts of the shoreline, with more easily accessible areas than others so repeated exposure is more likely at these focal locations. Further, we did not know where the residences of the SAC were, only their school location was considered in our models. Future studies, using a quantitative social study are required to improve our understanding of how exposure to snails along the shoreline is affected with distance from SAC school or residential living area^15^. For instance, other socio-economic factors including education, wealth, trade, or health could affect how often, where, and when SAC are exposed to snail habitats, and hence effect their *Schistosoma* transmission risk^39^.

We only had cross-sectional data for a single point in time (aggregate 2017–2019 data) due to having too small samples for each year. Due to this we were not able to consider the temporal interpretations of the model (e.g. equilibrium assumptions and transition durations). Ideally, in future studies, a longitudinal cohort study should be carried out to attain more data, where we recruit a panel of children and then follow them every year. Firstly, this would allow us to better understand the sensitivity and specificity of the tests used as they are repeated. Secondly, this would allow us to study how the dynamic of infection changes over time. In particular, it would provide the opportunity to study the effects of co-morbidities (e.g. HIV status) on the build-up or loss of immunity allowing us to understand the drivers of age-prevalence profiles. Similarly, a longitudinal study would allow us to study any effects of MDA that might be concomitant.

Another limitation of the study is that we fixed some parameters for identifiability purposes (i.e. Fig. 10). For instance, we assumed immunity to be under one day for age 6 SAC due to the optimiser difficulty in identifying omega against the other parameters, however estimating omega would be useful if we had more data on the children’s partial immunity to be able to find their actual immunity accumulated over time. Further, we could have explored immunity to be continuous with respect to age, however, this would have required a more complex partial differential equation setup. This would have concomitant implications for increased complexity of the numerical machinery required to solve these equations^40^. Kura et al*.*^41^, reported that in the presence of acquired immunity MDA programmes were less effective in decreasing the prevalence of infection compared to no acquired immunity^41^. Hence, the ability to estimate immunity within SAC could improve application of appropriate control methods. Further, we could have also accounted for treatment from MDA, which can affect the children’s infection status and load of infection (light/heavy) which was not considered in this study.

To improve how our model captures the dynamics of *Schistosoma* transmission, the identifiability of parameters and the significant statistical evidence of the model. Future work preferably using longitudinal collected data could be carried out using a Bayesian approach to look at the structure of the posterior very carefully and work out whether it’s the non-identifiability of parameters in the model that mean we can’t trust the Gaussian approximation that we need for calculating the CI in the way we designed our models. In additional, machine learning could be used to improve parameter estimated enhancing the model ability to capture complex transmission dynamics particularly in regions with significant variability^42^.

Furthermore, a limitation of the model was that we assumed the all-snail infection drops off by 100km, although this is a sensible assumption as it is unlikely SAC would travel more than 100km from the shoreline: this is a weakness of the model given our data. Another limitation of our study is we assumed that all snails are infected and snail’s infected status is assumed independent of contact with SAC, so presence of snail indicates exposure to *Schistosoma* infection and SAC are independent of the overall human-snail-schistosome lifecycle. This may not be the case in reality. However, in spite of these several limitations, our model provides insight into how proximity to snail abundance might affect the patterns of age prevalence in schools nearby to the shoreline.

## Conclusion

Our study reproduces the non-linear age-prevalence profiles by modelling the *Schistosoma* infection transmission dynamics with age-related immunity using cross-sectional parasitology and malacological data already collected and analysed. We find the best estimates values of the main determinants of *Schistosoma* risk with SAC at schools along the southern part of Lake Malawi. One of the main outcomes of our study is that school measured distance from the shoreline has no tangible effect on the SAC exposure. However, our dataset was limited by our assumptions of the model, size and single-time-point. We were able to a certain extent to reconstruct the age-infection profile using a simple state transmission model. However, further studies could be carried out using a longitudinal cohort study, which would be much more powerful than a single cross-sectional study to understand *Schistosoma* infection dynamics and how this affects SAC age-infection profiles and the implications on how control methods are applied.

## Supplementary Information

Supplementary Information 1.

Supplementary Information 2.

Supplementary Information 3.

## Data Availability

All code for this publication is accessible on Zenodo. 10.5281/zenodo.10410481. The primary data is provided in Supplementary Dataset S1 and S2.
