# Supplementary material for: Development of a dynamical model to enhance understanding of epidemiology of schistosomiasis in school-aged-children
Source: Sci Rep. 2025 Dec 22;15:45091. doi: 10.1038/s41598-025-32664-w (PMC12749467; doi:10.1038/s41598-025-32664-w)
Supplement: Supplementary file 3 — Supplementary Information 3. [file 41598_2025_32664_MOESM3_ESM.pdf]

Supplementary information

Construction of 200 prediction points along shoreline

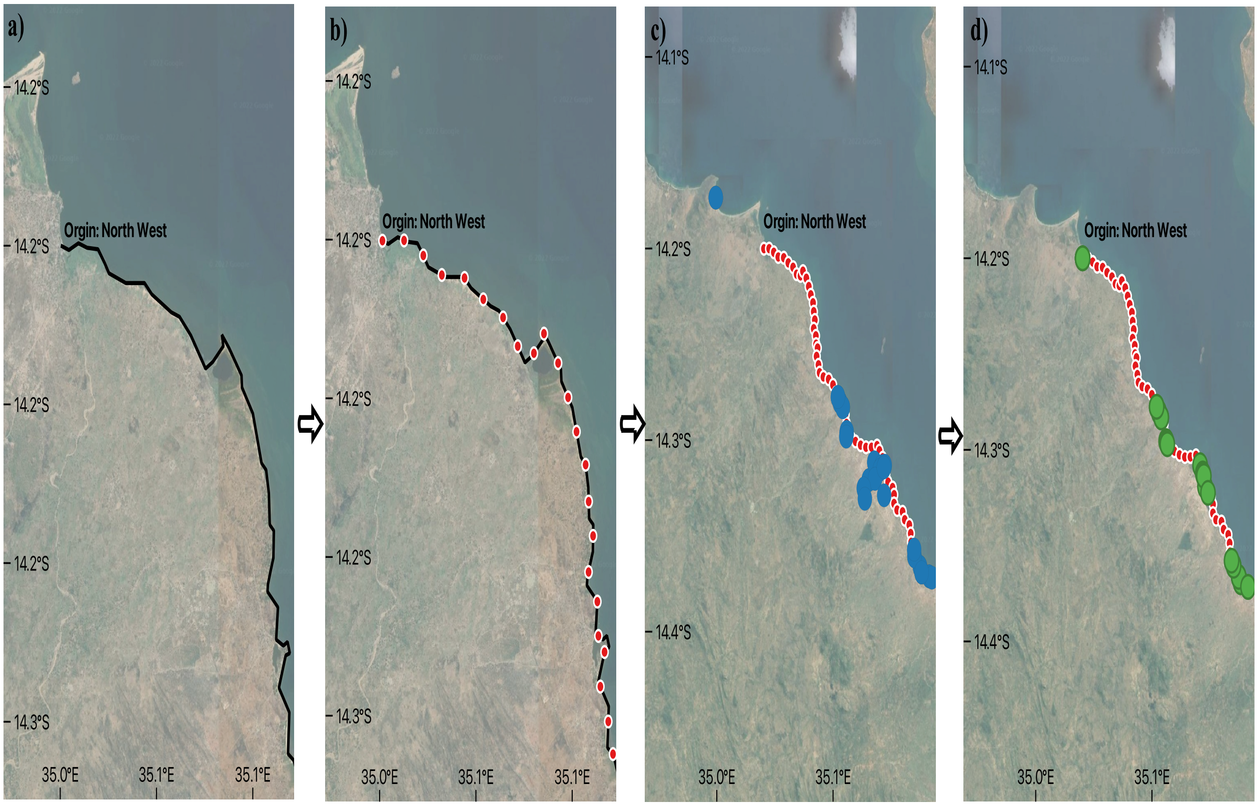

Figure S1: Flow diagram showing the stages for constructing the 200 predictions along the shoreline. **a)** A 2-D linestring was drawn by hand following the shoreline as shown by Google Satellite imagery, **b)** the linestring was re-sampled to 4000 equally spaced vertices and resampled them to 200 equally intervals (red dots), **c)** observed sampling site locations (blue dots), **d)** each observed sampling site location was snapped to its nearest vertex (green dots).

Log-likelihood profiles

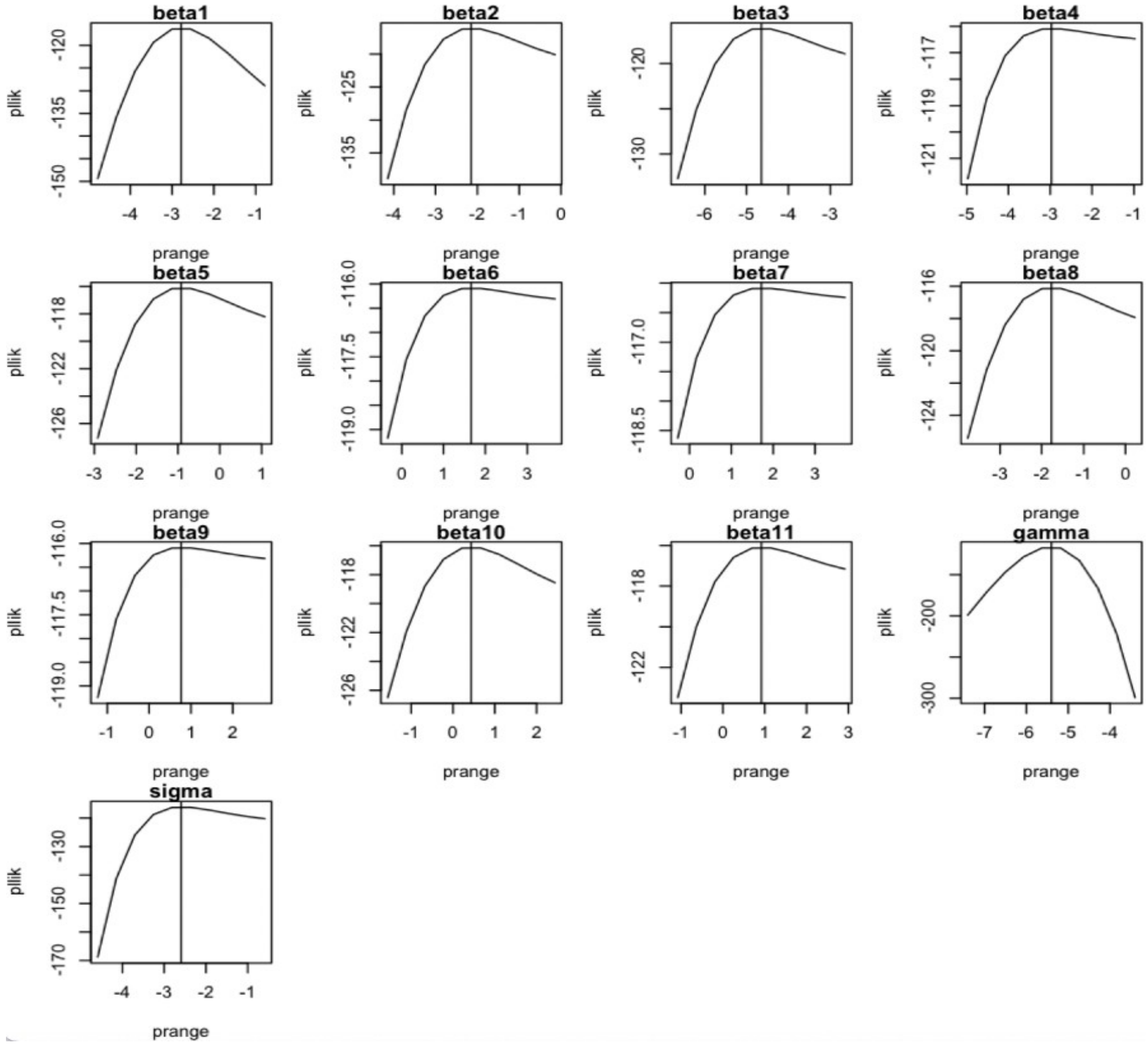

Figure S2: *Biomphalaria* sp. Multi-β_s with space effect model profile likelihood against fit of model.

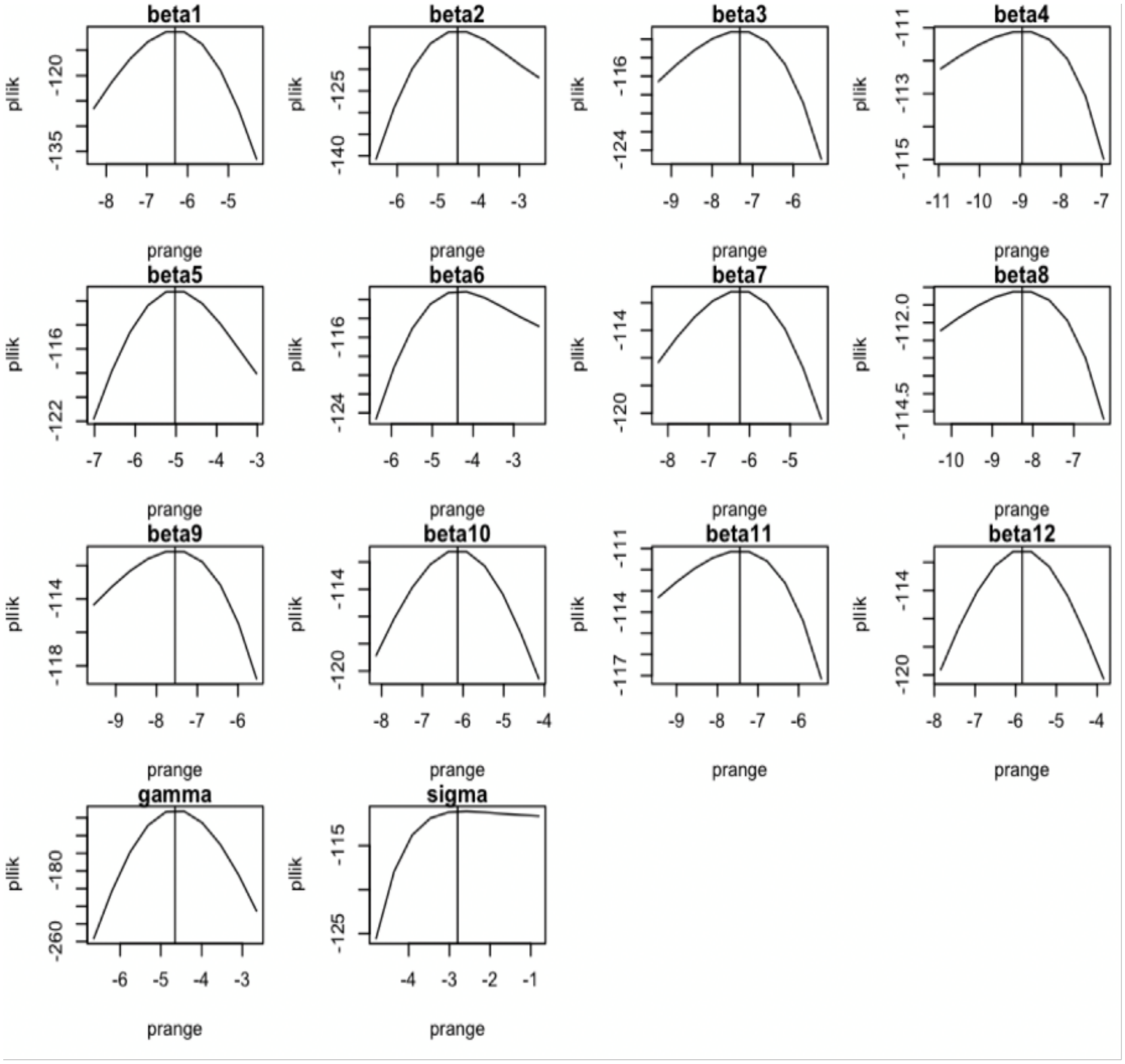

Figure S3: *Bulinus* sp. Multi-β_s with space effect model profile likelihood against fit of model

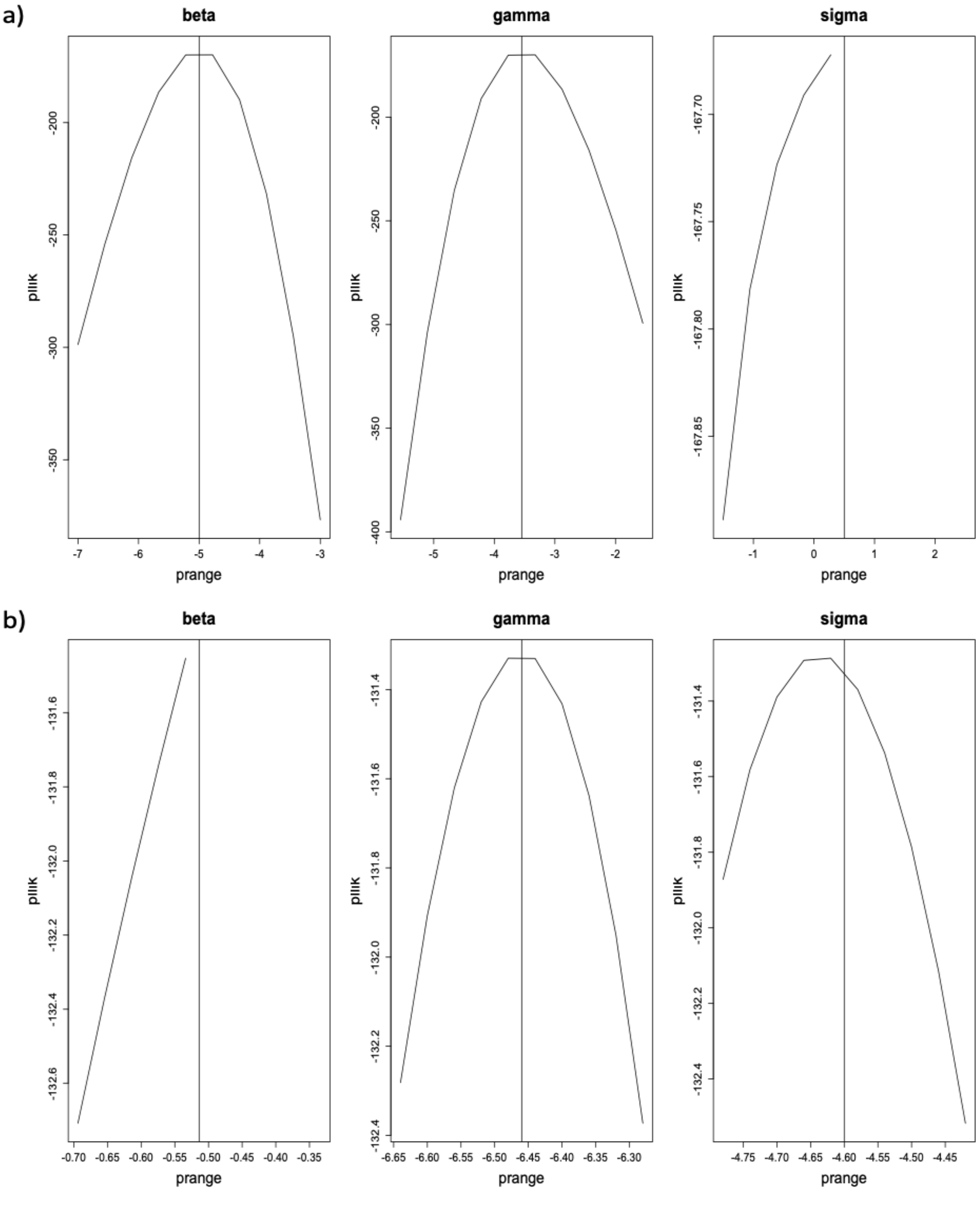

Figure S4: Single-β with space effect model profile likelihood against fit of model **a)** *Biomphalaria* sp. **b)** *Bulinus* spp.

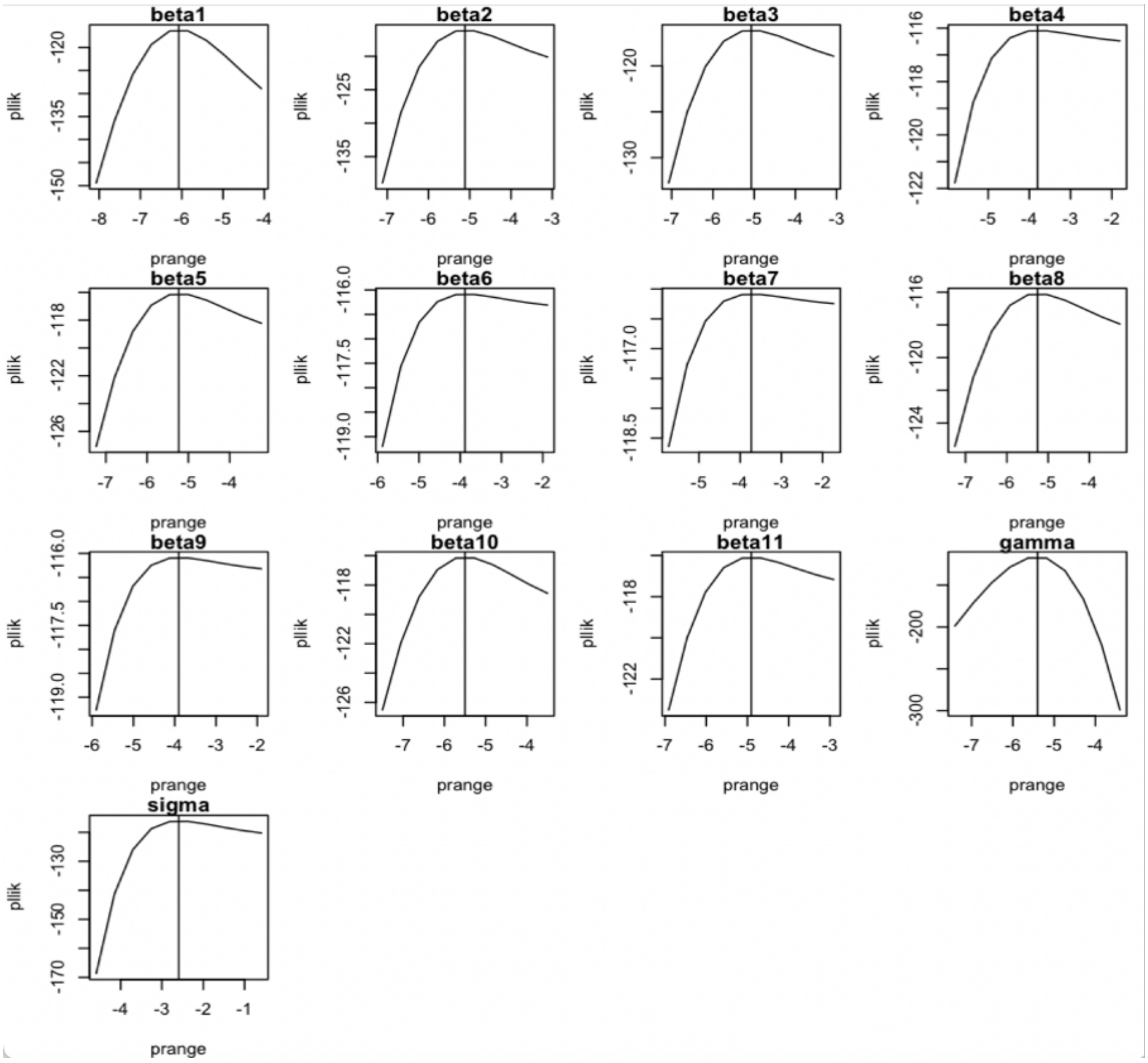

Figure S5: *Biomphalaria* sp. Multi-β_s with no space effect model profile likelihood against fit of model.

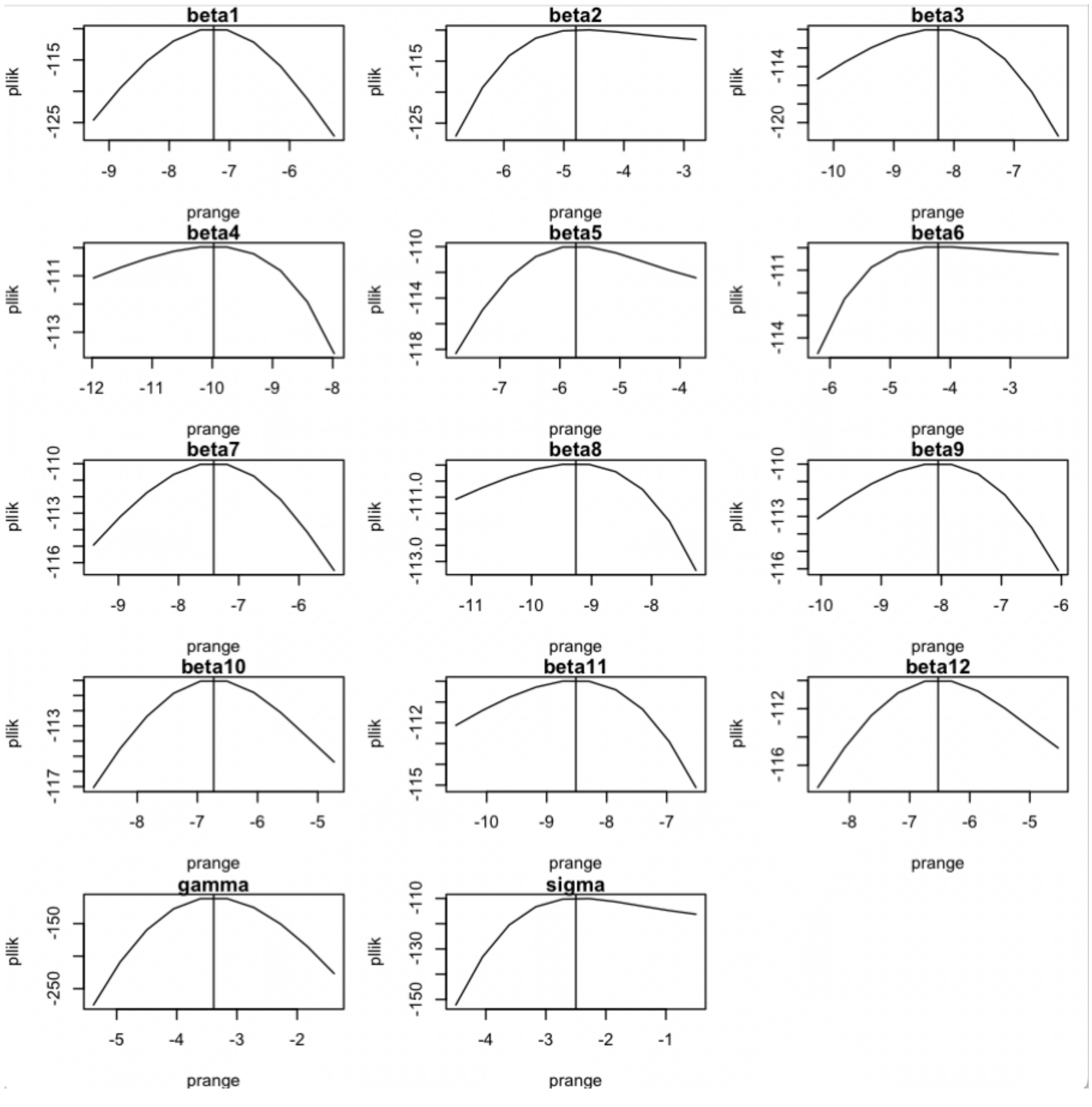

Figure S6: *Bulinus* sp. Multi-β_s with no space effect model profile likelihood against fit of model

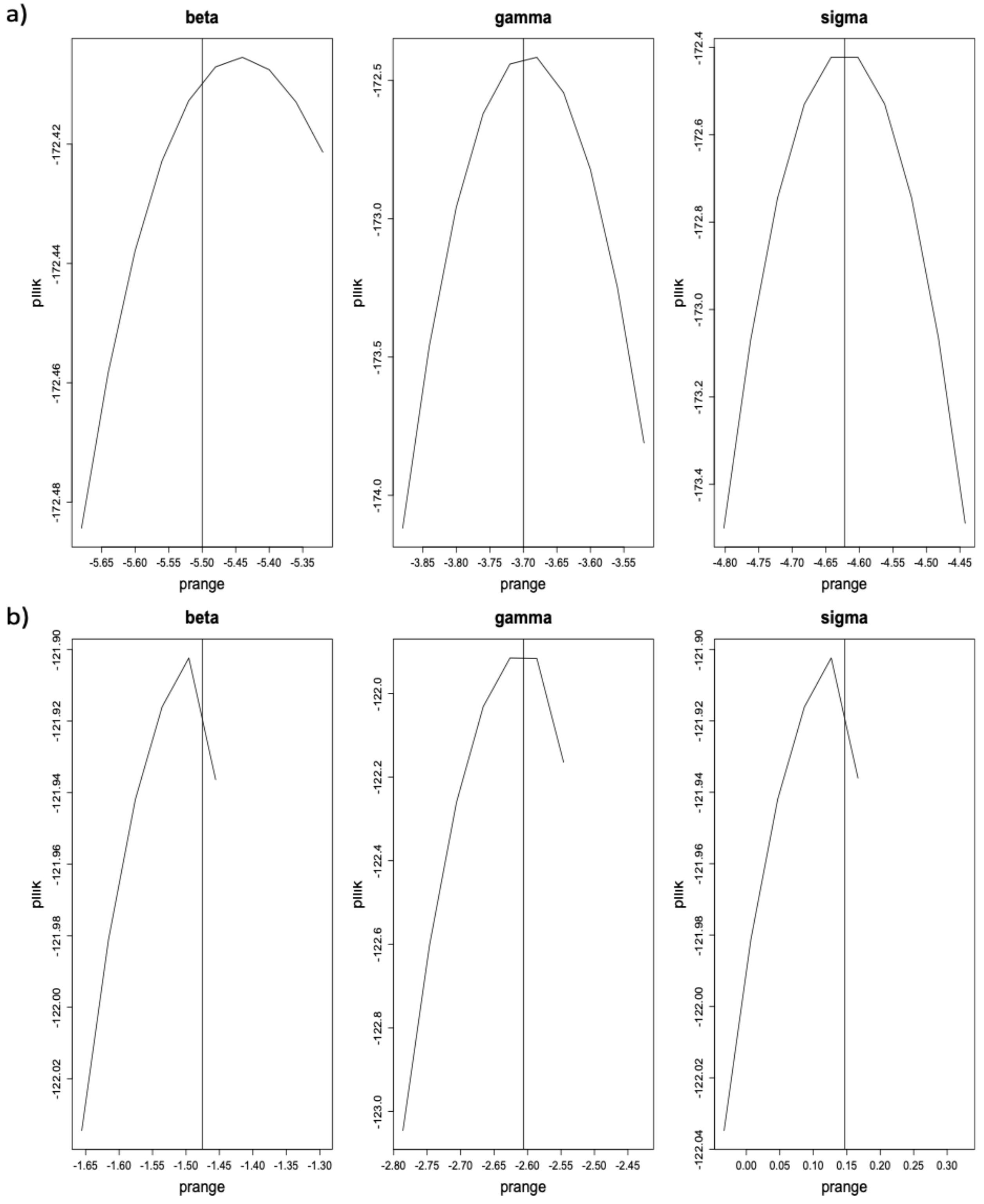

Figure S7: Single-β with no space effect model profile likelihood against fit of model **a)** *Biomphalaria* sp. **b)** *Bulinus* spp.

Sensitivity analysis

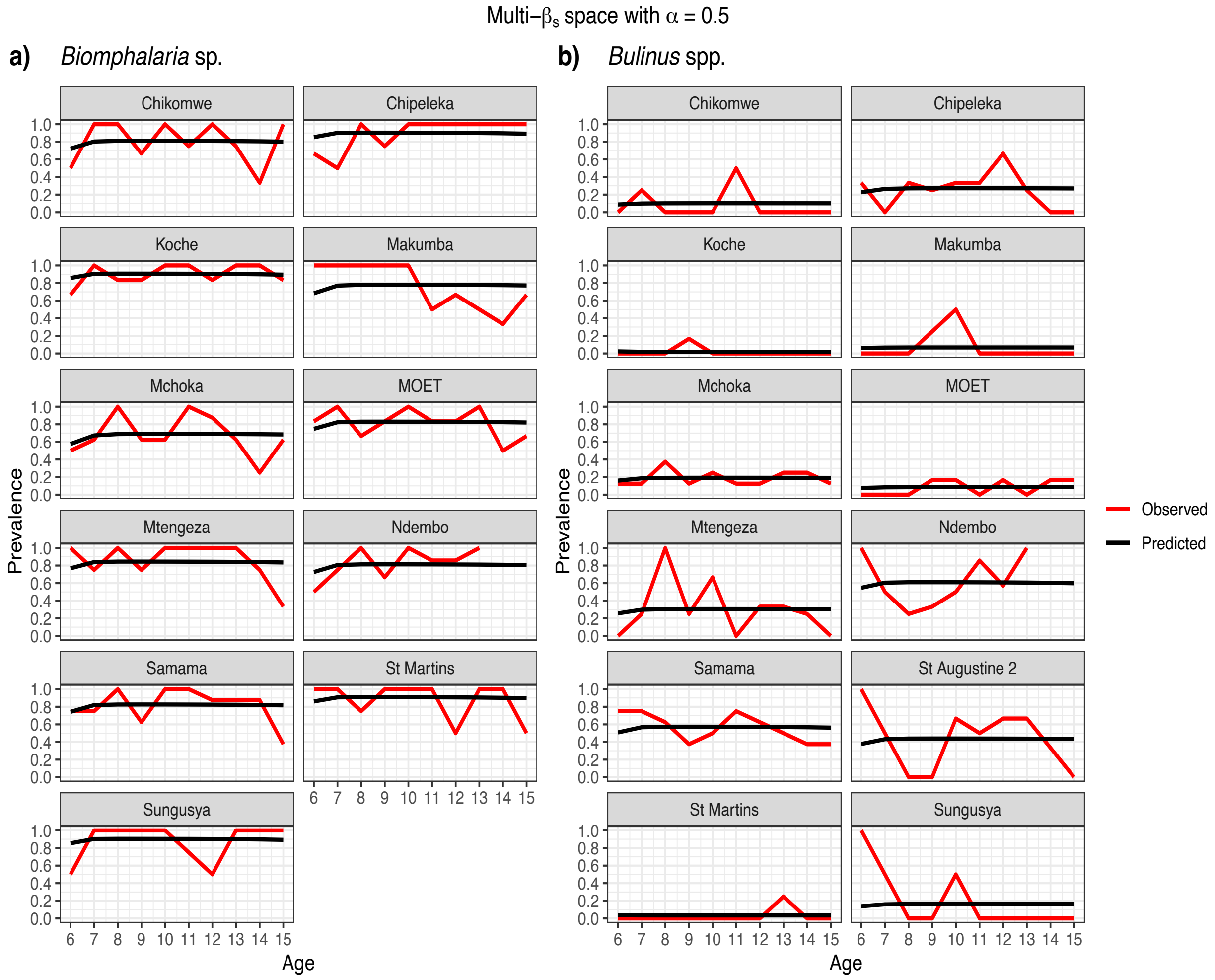

Figure S8: Prior *Schistosoma* infection prevalence of α = 0.05 for SAC age 6. Multi-β_s with space effect model optimisation prevalence prediction (black line) and observed prevalence (red line) against age of SAC carried out for each species **a)** *Biomphalaria* sp. **b)** *Bulinus* spp.

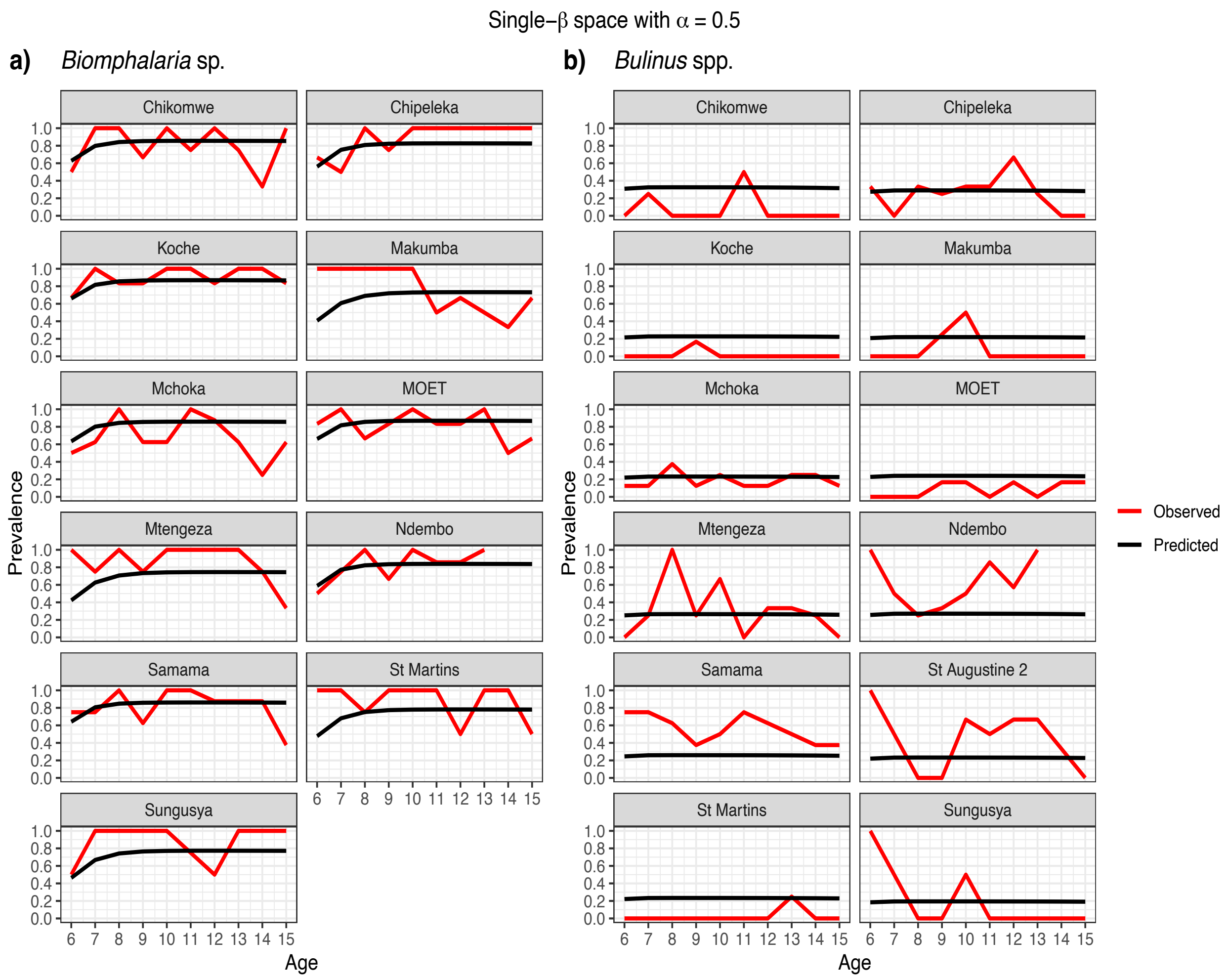

Figure S9: Prior *Schistosoma* infection prevalence of $\alpha = 0.05$ for SAC age 6. Single-β with space effect model optimisation prevalence prediction (black line) and observed prevalence (red line) against age of SAC carried out for each species **a)** *Biomphalaria* sp. **b)** *Bulinus* spp.

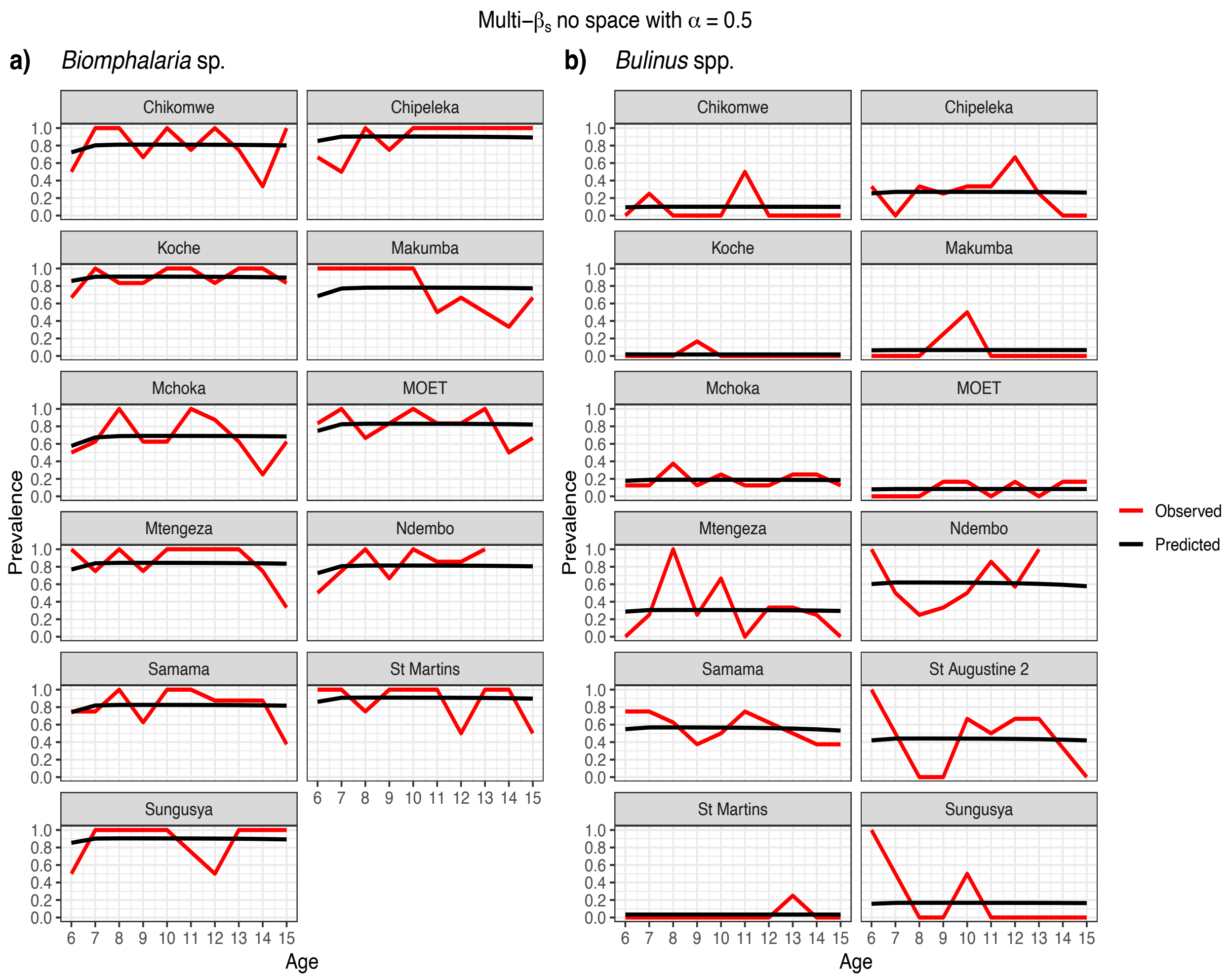

Figure S10: Prior *Schistosoma* infection prevalence of $\alpha = 0.05$ for SAC age 6. Multi-β_s with no space effect model optimisation prevalence prediction (black line) and observed prevalence (red line) against age of SAC carried out for each species **a)** *Biomphalaria* sp. **b)** *Bulinus* spp.

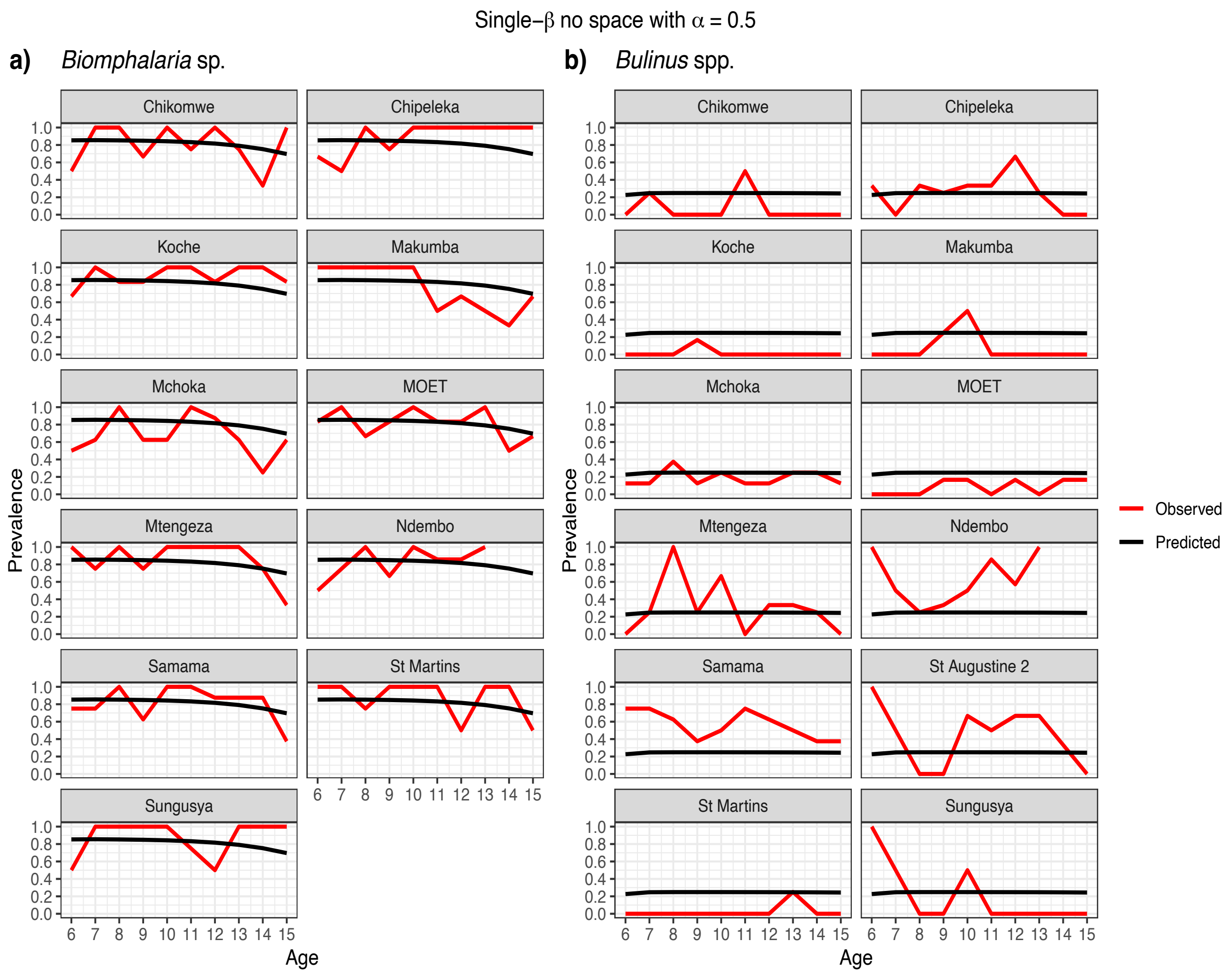

Figure S11: Prior *Schistosoma* infection prevalence of $\alpha = 0.05$ for SAC age 6. Single-β with no space effect model optimisation prevalence prediction (black line) and observed prevalence (red line) against age of SAC carried out for each species **a)** *Biomphalaria* sp. **b)** *Bulinus* spp.

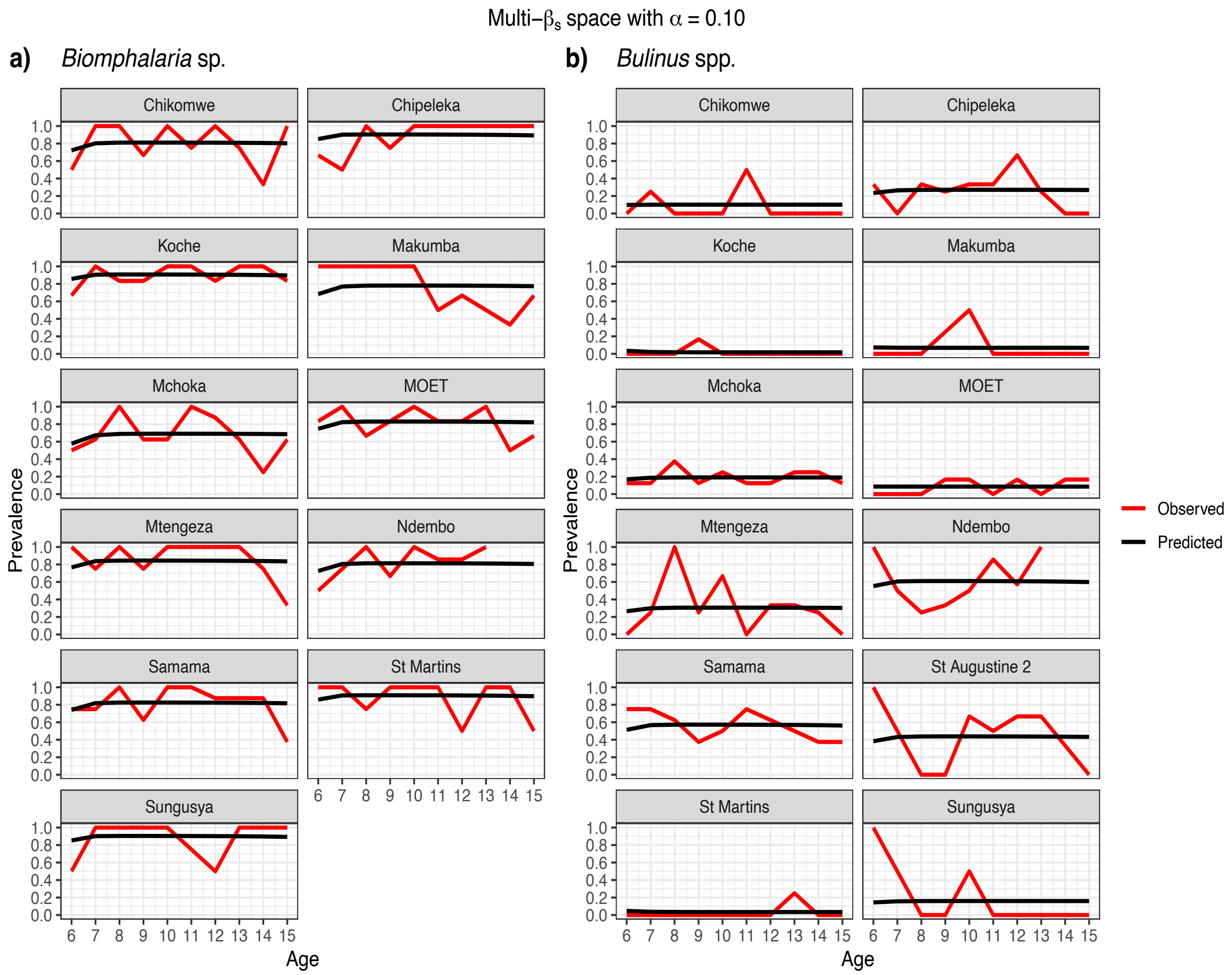

Figure S12: Prior *Schistosoma* infection prevalence of $\alpha = 0.10$ for SAC age 6. Multi-β_s with space effect model optimisation prevalence prediction (black line) and observed prevalence (red line) against age of SAC carried out for each species **a)** *Biomphalaria* sp. **b)** *Bulinus* spp.

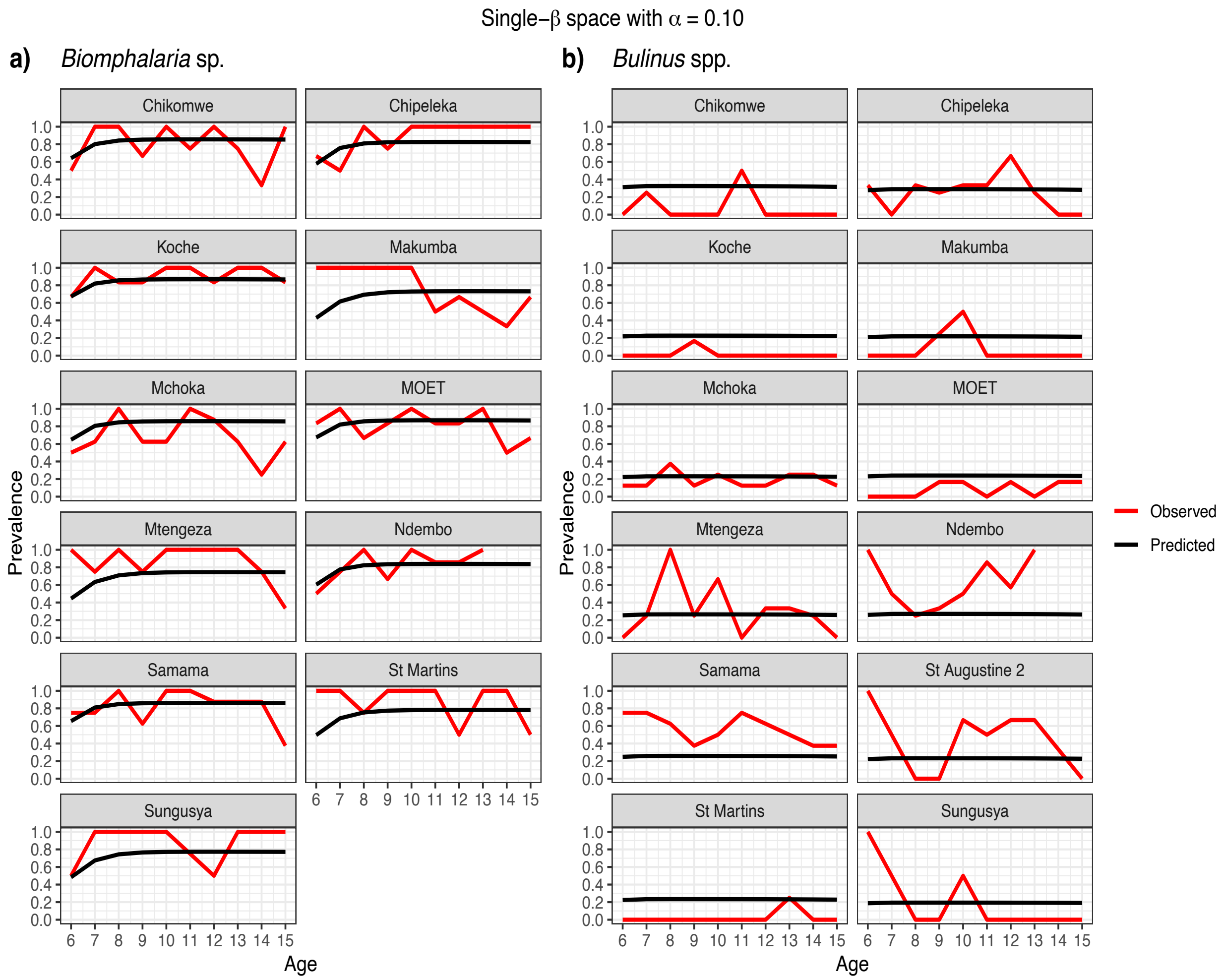

Figure S13: Prior *Schistosoma* infection prevalence of $\alpha = 0.10$ for SAC age 6. Single-β with space effect model optimisation prevalence prediction (black line) and observed prevalence (red line) against age of SAC carried out for each species **a)** *Biomphalaria* sp. **b)** *Bulinus* spp.

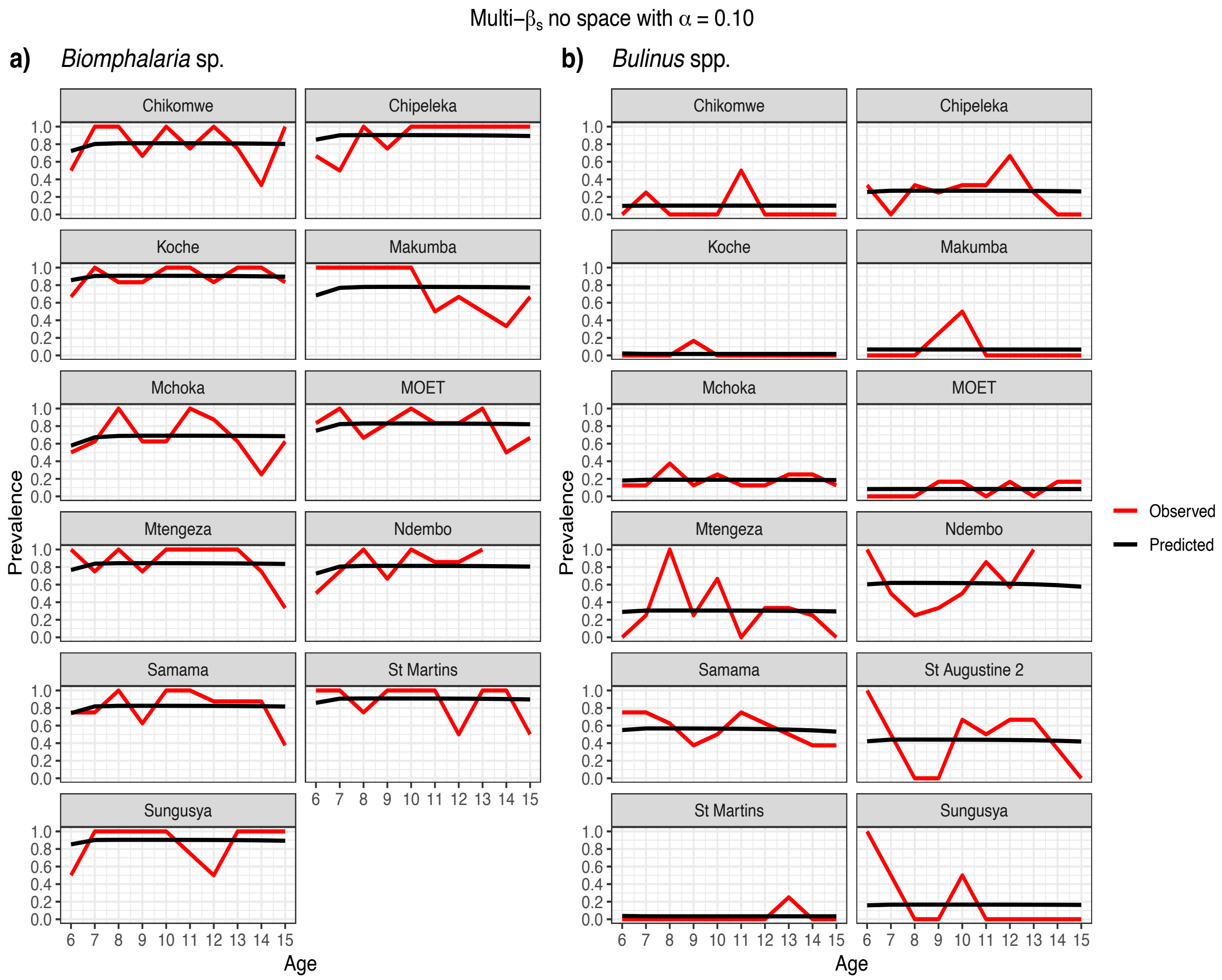

Figure S14: Prior *Schistosoma* infection prevalence of $\alpha = 0.10$ for SAC age 6. Multi-β_s with no space effect model optimisation prevalence prediction (black line) and observed prevalence (red line) against age of SAC carried out for each species **a)** *Biomphalaria* sp. **b)** *Bulinus* spp.

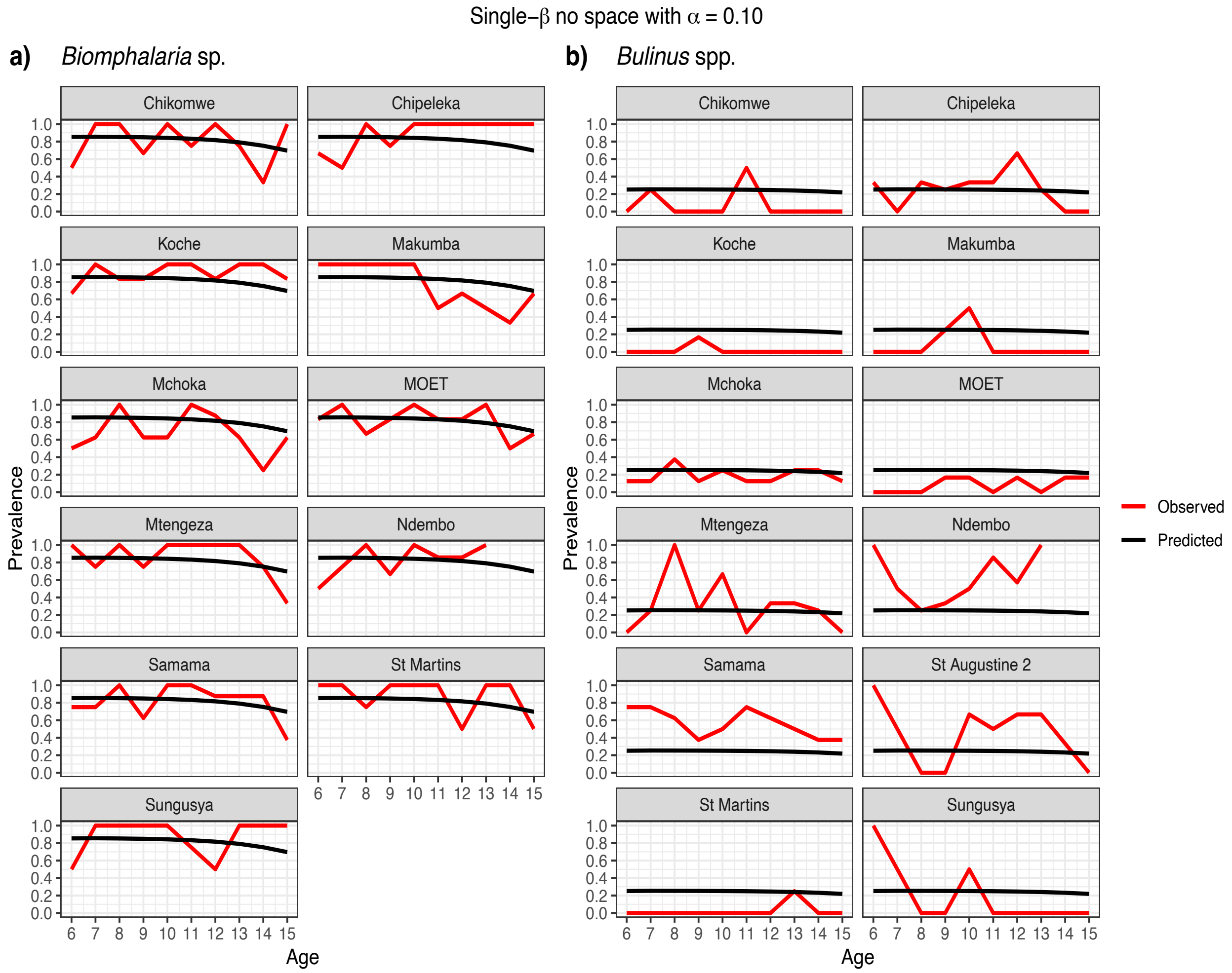

Figure S15: Prior *Schistosoma* infection prevalence of $\alpha = 0.10$ for SAC age 6. Single-β with no space effect model optimisation prevalence prediction (black line) and observed prevalence (red line) against age of SAC carried out for each species **a)** *Biomphalaria* sp. **b)** *Bulinus* spp.

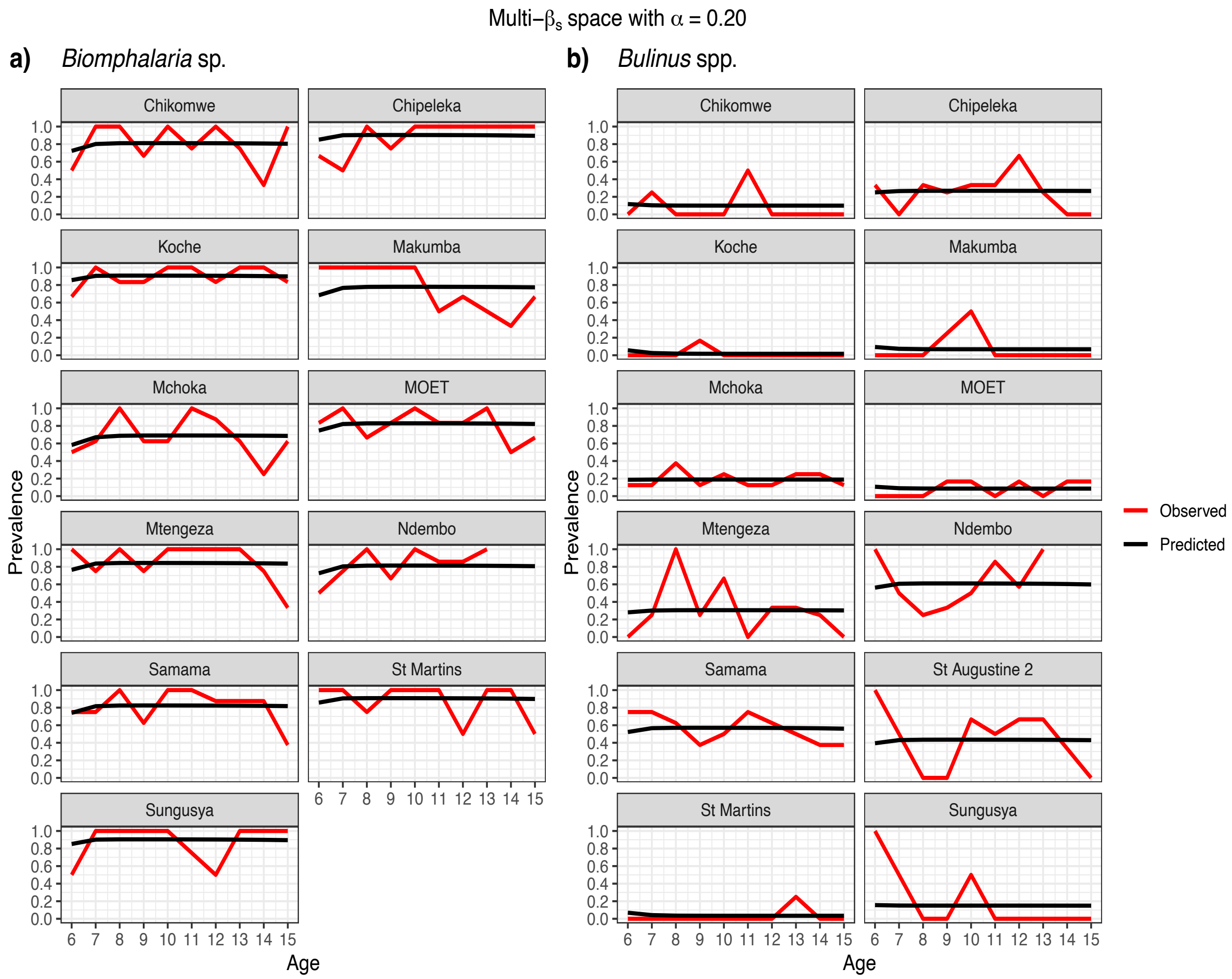

Figure S16: Prior *Schistosoma* infection prevalence of $\alpha = 0.20$ for SAC age 6. Multi-β_s with space effect model optimisation prevalence prediction (black line) and observed prevalence (red line) against age of SAC carried out for each species **a)** *Biomphalaria* sp. **b)** *Bulinus* spp.

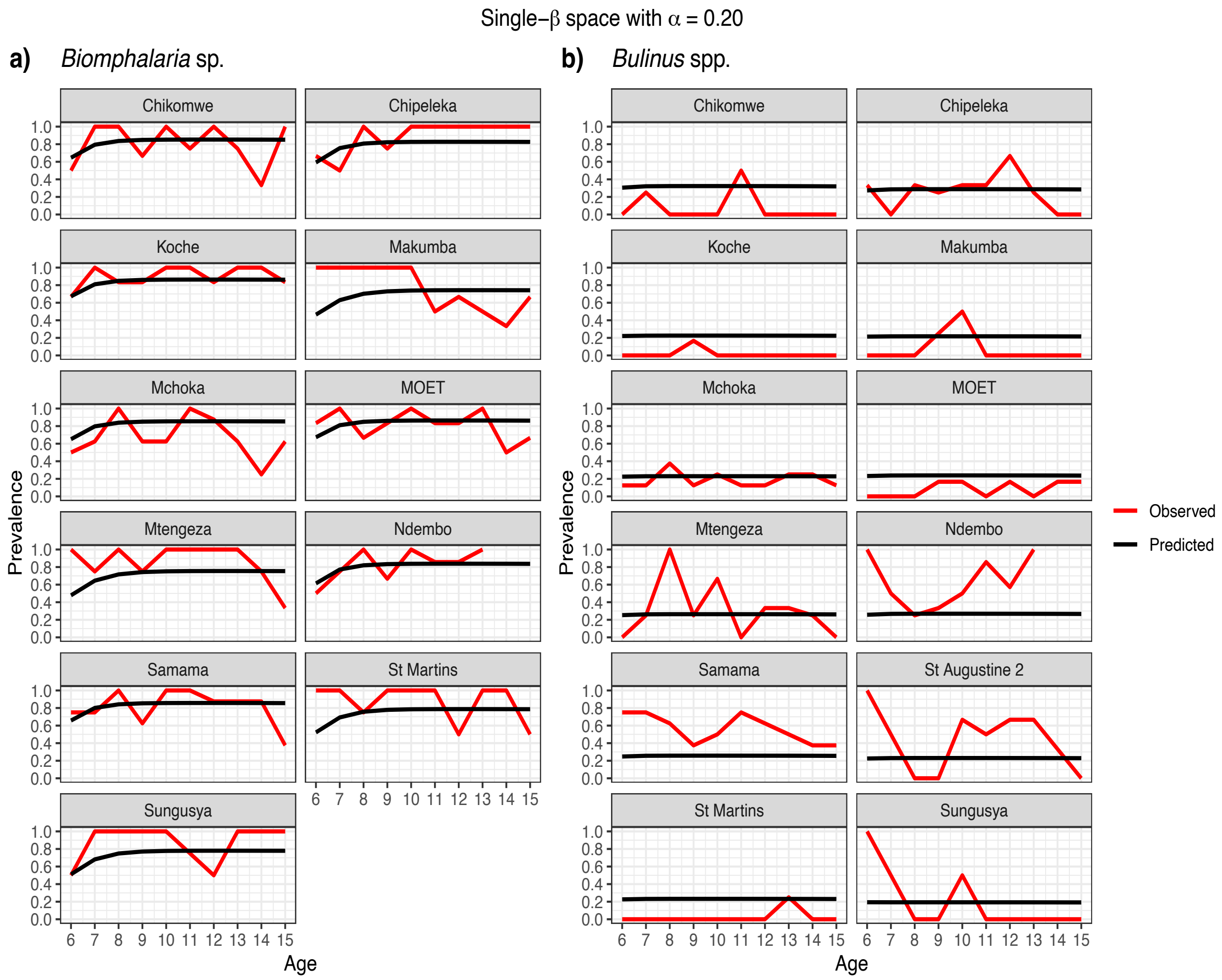

Figure S17: Prior *Schistosoma* infection prevalence of $\alpha = 0.20$ for SAC age 6. Single-β with space effect model optimisation prevalence prediction (black line) and observed prevalence (red line) against age of SAC carried out for each species **a)** *Biomphalaria* sp. **b)** *Bulinus* spp.

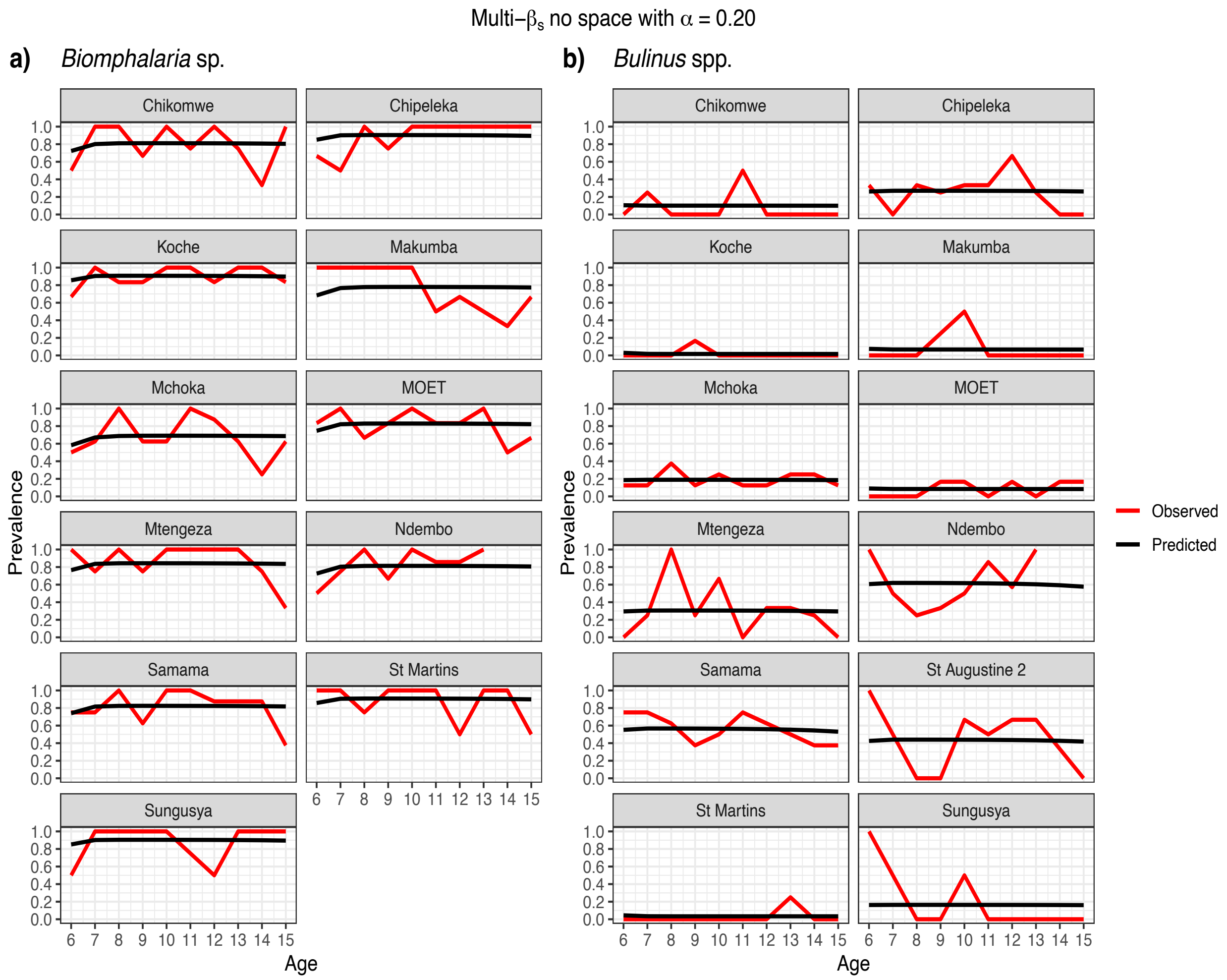

Figure S18: Prior *Schistosoma* infection prevalence of α = 0.20 for SAC age 6. Multi-β_s with no space effect model optimisation prevalence prediction (black line) and observed prevalence (red line) against age of SAC carried out for each species **a)** *Biomphalaria* sp. **b)** *Bulinus* spp.

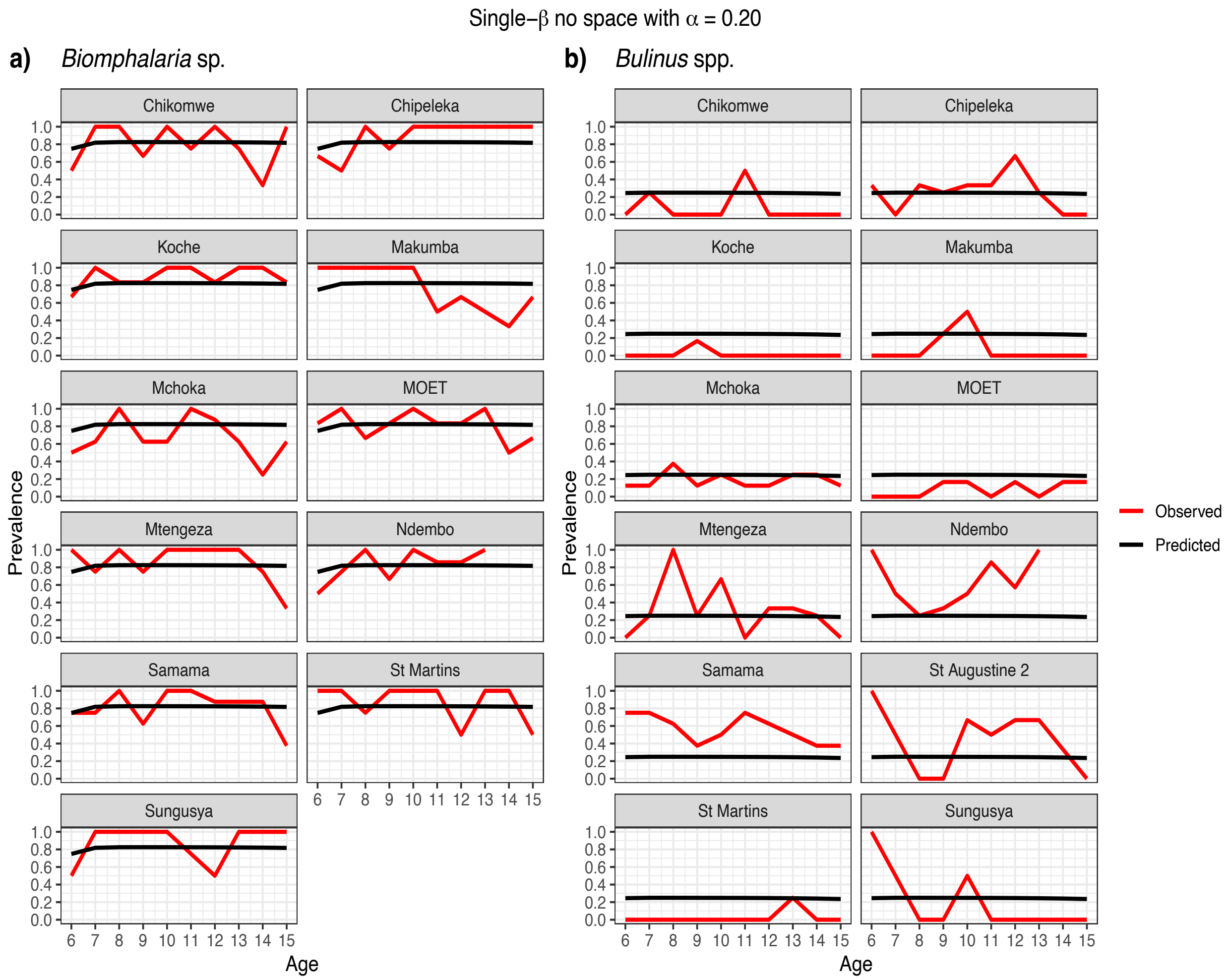

Figure S19: Prior *Schistosoma* infection prevalence of $\alpha = 0.20$ for SAC age 6. Single-β with no space effect model optimisation prevalence prediction (black line) and observed prevalence (red line) against age of SAC carried out for each species **a)** *Biomphalaria* sp. **b)** *Bulinus* spp.

Table S1: Prior *Schistosoma* infection prevalence of $\alpha = 0.05$ for SAC age 6. Parameter estimates for multi-β_s spatial model for each species

Multi-β_s space with $\alpha = 0.05$

<table>
<tr><th></th><th colspan="3">Biomphalaria
AIC=258</th><th>MSE</th><th colspan="3">Bulinus
AIC=250</th><th>MSE</th></tr>
<tr><th>Parmeters/Schools [CI]</th><th>log β</th><th>log σ</th><th>log γ</th><th></th><th>log β</th><th>log σ</th><th>log γ</th><th></th></tr>
<tr><td>Mchoka</td><td>-2.86
[-5.28, -0.438]</td><td rowspan="4">-2.66
[-9.01, 3.69]</td><td rowspan="4">-5.48
[-7.64, -3.31]</td><td>0.443</td><td>-6.31
[-8.47, -4.16]</td><td rowspan="12">-2.80
[-8,20, 2.60]</td><td rowspan="12">-4.65
[-6.65, 2.65]</td><td>0.0668</td></tr>
<tr><td>Samama</td><td>-2.23
[-5.77, 1.32]</td><td>0.340</td><td>-4.52
[-7.62, -1.43]</td><td>0.251</td></tr>
<tr><td>MOET</td><td>-4.73
[-8.39, -1.07]</td><td>0.253</td><td>-7.32
[-9.51, -5.12]</td><td>0.0675</td></tr>
<tr><td>Koche</td><td>-3.06
[-12.8, 6.67]</td><td>0.102</td><td>-8.95
[-11.8, -6.12]</td><td>0.0253</td></tr>
<tr><td>St Augustine 2</td><td></td><td></td><td></td><td></td><td>-5.03
[-7.63, -2.42]</td><td>1.14</td></tr>
<tr><td>Ndembo</td><td>-1.00
[-4.44, 2.43]</td><td rowspan="7"></td><td rowspan="7"></td><td>0.185</td><td>-4.38
[-7.79, -0.974]</td><td>0.653</td></tr>
<tr><td>Sungusya</td><td>1.58
[-7.69, 10.8]</td><td>0.379</td><td>-6.28
[-8.64, -3.91]</td><td>1.16</td></tr>
<tr><td>St Martins</td><td>1.62
[-9.27, 12.5]</td><td>0.420</td><td>-8.26
[-11.1, -5.46]</td><td>0.0573</td></tr>
<tr><td>Chikomwe</td><td>-1.85
[-5.24, 1.54]</td><td>0.487</td><td>-7.55
[-9.92, -5.18]</td><td>0.261</td></tr>
<tr><td>Chipeleka</td><td>0.679
[-8.55, 9.91]</td><td>0.290</td><td>-6.14
[-8.47, -3.82]</td><td>0.396</td></tr>
<tr><td>Makumba</td><td>0.356
[-2.70, 3.41]</td><td>0.673</td><td>-7.45
[-9..91,- 4.98]</td><td>0.256</td></tr>
<tr><td>Mtengeza</td><td>0.833
[-3.44, 5.11]</td><td>0.453</td><td>-5.84
[-8.19,-3.49]</td><td>0.874</td></tr>
</table>

Table S2: Prior *Schistosoma* infection prevalence of $\alpha = 0.05$ for SAC age 6. Parameter estimates for single-β spatial model for each species

Single-β space with $\alpha = 0.05$

	Biomphalaria AIC=275			MSE	*Bulinus* AIC=341			MSE
**Parmeters/Schools [CI]**	$\log\beta$	$\log\sigma$	$\log\gamma$		$\log\beta$	$\log\sigma$	$\log\gamma$	
**Mchoka**	-1.00 [-1.67, -0.328]	-4.96 [-5.49, -4.42]	-6.86 [-7.45, -6.28]	0.677	-5.00 [-10.4, 0.350]	0.500 [-12.7, 13.7]	-3.55 [-8.84, 1.75]	0.0861
**Samama**				0.366				1.16
**MOET**				0.313				0.308
**Koche**				0.107				0.460
**St Augustine 2**								1.49
**Ndembo**				0.121				1.63
**Sungusya**				0.516				1.11
**St Martins**				0.777				0.483
**Chikomwe**				0.474				0.859
**Chipeleka**				0.298				0.400
**Makumba**				1.03				0.456
**Mtengeza**				0.865				0.911

Table S3: Prior *Schistosoma* infection prevalence of $\alpha = 0.05$ for SAC age 6. Parameter estimates for multi-β_s no space model for each species

Multi-β_s no space with $\alpha = 0.05$

	Biomphalaria AIC=258			MSE	Bulinus AIC=248			MSE
**Parameters/Schools [CI]**	$\log \beta$	$\log \sigma$	$\log \gamma$		$\log \beta$	$\log \sigma$	$\log \gamma$	
**Mchoka**	-6.14 [-8.56, -3.73]	-2.67 [-8.90, 3.55]	-5.48 [-7.64, -3.31]	0.443	-7.26 [-12.5, -2.02]	-2.50 [-8.51, 3.51]	-3.39 [-8.61, 1.84]	0.0686
**Samama**	-5.19 [-8.73, -1.65]			0.340	-4.80 [-11.0, 1.44]			0.214
**MOET**	-5.14 [-8.80, -1.49]			0.253	-8.26 [-13.5, -3.01]			0.0688
**Koche**	-3.87 [-13.7, 5.94]			0.102	-9.97 [-15.5, -4.43]			0.0251
**St Augustine 2**					-5.73 [-11.3, -0.177]			1.08
**Ndembo**	-5.30 [-8.73, -1.87]			0.185	-4.20 [-12.1, 3.74]			0.623
**Sungusya**	-3.95 [-13.3, 5.39]			0.379	-7.42 [-12.8, -2.03]			1.13
**St Martins**	-3.81 [-14.8, 7.21]			0.420	-9.26 [-14.8, -3.70]			0.0570
**Chikomwe**	-5.33 [-8.71, -1.95]			0.487	-8.05 [-13.4, -2.69]			0.262
**Chipeleka**	-3.96 [-13.3, 5.36]			0.290	-6.73 [-12.1, -1.37]			0.390
**Makumba**	-5.57 [-8.63, -2.52]			0.673	-8.51 [-13.9, -3.11]			0.256
**Mtengeza**	-4.98 [-9.26, -0.714]			0.453	-6.53 [-11.9, -1.12]			0.885

Table S4: Prior *Schistosoma* infection prevalence of $\alpha = 0.05$ for SAC age 6. Parameter estimates for single-β no space model for each species

Single-β no space with $\alpha = 0.05$

	Biomphalaria AIC=250			MSE	*Bulinus* AIC=351			MSE
**Parmeters/Schools [CI]**	$\log\beta$	$\log\sigma$	$\log\gamma$		$\log\beta$	$\log\sigma$	$\log\gamma$	
**Mchoka**	-1.48 [-4.87, 1.92]	0.148 [-3.24, 3.54]	-2.61 [-2.85. -2.36]	0.613	-5.50 [-17.5, 6.49]	-4.63 [-16.8, 7.56]	-3.70 [-12.2, 4.85]	0.101
**Samama**				0.275				1.24
**MOET**				0.190				0.329
**Koche**				0.233				0.546
**St Augustine 2**								1.44
**Ndembo**				0.262				1.78
**Sungusya**				0.519				1.15
**St Martins**				0.372				0.540
**Chikomwe**				0.535				0.542
**Chipeleka**				0.476				0.389
**Makumba**				0.506				0.541
**Mtengeza**				0.326				0.926

Table S5: Prior *Schistosoma* infection prevalence of $\alpha = 0.10$ for SAC age 6. Parameter estimates for multi-β_s spatial model for each species

Multi-β_s space with $\alpha = 0.10$

	Biomphalaria AIC=258			MSE	*Bulinus* AIC=250			MSE
**Parmeters/Schools [CI]**	$\log \beta$	$\log \sigma$	$\log \gamma$		$\log \beta$	$\log \sigma$	$\log \gamma$	
**Mchoka**	-2.94 [-5.26, -0.618]			0.443	-6.32 [-8.62, -4.02]			0.0676
**Samama**	-2.31 [-5.71, 1.09]	-2.74 [-8.83, 3.34]	-5.56 [-7.62, -3.49]	0.340	-4.53 [-7.80, -1.25]			0.248
**MOET**	-4.81 [-8.33, -1.30]			0.254	-7.32 [-9.63, -5.00]	-2.80 [-8.51, 2.91]	-4.65 [-6.79, -2.51]	0.0676
**Koche**	-3.14 [-12.5, 6.19]			0.102	-8.95 [-11.9, -5.98]			0.0260
**St Augustine 2**					-5.03 [-7.79, -2.27]			1.13
**Ndembo**	-1.08 [-4.38 2.22]			0.185	-4.38 [-7.99, -0.775]			0.648
**Sungusya**	1.50 [-7.43, 10.4]			0.378	-6.32 [-8.91, -3.73]			1.15
**St Martins**	1.53 [-8.85, 11.9]			0.421	-8.26 [-11.2 -5.36]			0.0584
**Chikomwe**	-1.93 [-5.19, 1.33]			0.487	-7.55 [-10.1, -5.05]			0.263
**Chipeleka**	0.604 [-8.33, 9.53]			0.289	-6.15 [-8.62, -3.68]			0.394
**Makumba**	0.273 [-2.67, 3.21]			0.674	-7.45 [-10.0,- 4.87]			0.257
**Mtengeza**	0.748 [-3.35, 4.85]			0.454	-5.84 [-8.33,-3.36]			0.879

Table S6: Prior *Schistosoma* infection prevalence of $\alpha = 0.10$ for SAC age 6. Parameter estimates for single-β spatial model for each species

Single-β space with $\alpha = 0.10$

	Biomphalaria AIC=275			MSE	*Bulinus* AIC=341			MSE
**Parmeters/Schools [CI]**	$\log\beta$	$\log\sigma$	$\log\gamma$		$\log\beta$	$\log\sigma$	$\log\gamma$	
**Mchoka**	-1.00 [-1.67, -0.328]	-4.96 [-5.49, -4.42]	-6.86 [-7.45, -6.28]	0.682	-5.00 [-13.8, 3.80]	0.500 [-13.9, 14.9]	-3.55 [-12.2, 5.14]	0.0866
**Samama**				0.364				1.16
**MOET**				0.308				0.309
**Koche**				0.106				0.460
**St Augustine 2**								1.49
**Ndembo**				0.124				1.63
**Sungusya**				0.509				1.11
**St Martins**				0.753				0.483
**Chikomwe**				0.476				0.860
**Chipeleka**				0.297				0.399
**Makumba**				0.995				0.456
**Mtengeza**				0.837				0.913

Table S7: Prior *Schistosoma* infection prevalence of $\alpha = 0.10$ for SAC age 6. Parameter estimates for multi-β_s no space model for each species

Multi-β_s no space with $\alpha = 0.10$

	Biomphalaria AIC=258			MSE	Bulinus AIC=248			MSE
**Parameters/Schools [CI]**	$\log\beta$	$\log\sigma$	$\log\gamma$		$\log\beta$	$\log\sigma$	$\log\gamma$	
**Mchoka**	-6.22 [-8.55, -3.90]	-2.74 [-8.80, 3.32]	-5.56 [-7.63, -3.48]	0.443	-7.26 [-12.5, -1.98]	-2.50 [-8.56,3.57]	-3.38 [-8.64, 1.88]	0.0690
**Samama**	-5.27 [-8.67, -1.87]			0.340	-4.80 [-11.0, 1.44]			0.214
**MOET**	-5.23 [-8.74, -1.71]			0.254	-8.26 [-13.5, -2.97]			0.0694
**Koche**	-3.96 [-13.3, 5.38]			0.102	-9.97 [-15.5, -4.40]			0.0252
**St Augustine 2**					-5.73 [-11.3, -0.144]			1.07
**Ndembo**	-5.38 [-8.69, -2.08]			0.185	-4.19 [-12.1, 3.73]			0.621
**Sungusya**	-4.04 [-13.0, 4.89]			0.378	-7.43 [-12.9, -1.94]			1.12
**St Martins**	-3.91 [-14.3, 6.49]			0.421	-9.26 [-14.9, -3.67]			0.0573
**Chikomwe**	-5.41 [-8.67, -2.15]			0.487	-8.05 [-13.5, -2.65]			0.262
**Chipeleka**	-4.05 [-12.9, 4.89]			0.289	-6.73 [-12.1, -1.33]			0.389
**Makumba**	-5.66 [-8.60, -2.72]			0.674	-8.51 [-13.9, -3.07]			0.257
**Mtengeza**	-5.07 [-9.17, -0.971]			0.454	-6.52 [-12.0 -1.09]			0.887

Table S8: Prior *Schistosoma* infection prevalence of $\alpha = 0.10$ for SAC age 6. Parameter estimates for single-β no space model for each species

Single-β no space with $\alpha = 0.10$

	Biomphalaria AIC=250			MSE	*Bulinus* AIC=NA			MSE
**Parmeters/Schools [CI]**	$\log\beta$	$\log\sigma$	$\log\gamma$		$\log\beta$	$\log\sigma$	$\log\gamma$	
**Mchoka**	-1.48 [-4.80, 1.85]	0.148 [-3.17, 3.47]	-2.61 [-2.84. -2.37]	0.614	NA	NA	NA	NA
**Samama**				0.275				
**MOET**				0.190				
**Koche**				0.233				
**St Augustine 2**								
**Ndembo**				0.262				
**Sungusya**				0.519				
**St Martins**				0.372				
**Chikomwe**				0.535				
**Chipeleka**				0.476				
**Makumba**				0.506				
**Mtengeza**				0.326				

Table S9: Prior *Schistosoma* infection prevalence of $\alpha = 0.20$ for SAC age 6. Parameter estimates for multi-β_s spatial model for each species

Multi-β_s space with $\alpha = 0.20$

	Biomphalaria AIC=259			MSE	*Bulinus* AIC=250			MSE
**Parmeters/Schools [CI]**	$\log\beta$	$\log\sigma$	$\log\gamma$		$\log\beta$	$\log\sigma$	$\log\gamma$	
**Mchoka**	-3.09 [-5.31, -0.883]	-2.89 [-8.66, 2.88]	-5.71 [-7.67, -3.75]	0.445	-6.33 [-8.81, -3.85]	-2.80 [-9.26, 3.66]	-4.65 [-6.95 -2.35]	0.0668
**Samama**	-2.47 [-5.69, 0.747]			0.341	-4.54 [-8.08, -0.995]			0.242
**MOET**	-4.98 [-8.31, -1.64]			0.255	-7.32 [-9.78, -4.85]			0.0745
**Koche**	-3.31 [-12.1, 5.44]			0.101	-8.96 [-12.1, -5.77]			0.0282
**St Augustine 2**					-5.04 [-8.01, -2.07]			1.11
**Ndembo**	-1.24 [-4.38 1.91]			0.185	-4.38 [-8.29, -0.467]			0.639
**Sungusya**	1.34 [-7.08 9.75]			0.377	-6.40 [-9.34, -3.46]			1.11
**St Martins**	1.34 [-8.30, 11.0]			0.423	-8.26 [-11.3 -5.25]			0.0614
**Chikomwe**	-2.08 [-5.19, 1.03]			0.488	-7.57 [-10.3, -4.86]			0.265
**Chipeleka**	0.442 [-8.01, 8.89]			0.287	-6.16 [-8.81, -3.51]			0.391
**Makumba**	0.110 [-2.70, 2.92]			0.676	-7.45 [-10.2,- 4.72]			0.261
**Mtengeza**	0.578 [-3.30, 4.46]			0.456	5.84 [-8.51,-3.18]			0.888

Table S10: Prior *Schistosoma* infection prevalence of $\alpha = 0.20$ for SAC age 6. Parameter estimates for single-β spatial model for each species

Single-β space with $\alpha = 0.20$

	Biomphalaria AIC=271			MSE	*Bulinus* AIC=341			MSE
**Parmeters/Schools [CI]**	log β	log σ	log γ		log β	log σ	log γ	
**Mchoka**	-1.00 [-1.75, -0.253]	-5.13 [-5.74, -4.52]	-7.00 [-7.68, -6.32]	0.673	-6.00 [-14.6, 2.61]	0.500 [-12.8, 11.8]	-4.53 [-13.1, 4.00]	0.0867
**Samama**				0.361				1.17
**MOET**				0.305				0.307
**Koche**				0.114				0.459
**St Augustine 2**								1.49
**Ndembo**				0.127				1.64
**Sungusya**				0.492				1.10
**St Martins**				0.718				0.481
**Chikomwe**				0.479				0.858
**Chipeleka**				0.293				0.401
**Makumba**				0.951				0.457
**Mtengeza**				0.783				0.917

Table S11: Prior *Schistosoma* infection prevalence of $\alpha = 0.20$ for SAC age 6. Parameter estimates for multi-β_s no space model for each species

Multi-β_S no space with $\sigma = 0.20$

	Biomphalaria AIC=259			MSE	Bulinus AIC=248			MSE
**Parameters/Schools [CI]**	$\log\beta$	$\log\sigma$	$\log\gamma$		$\log\beta$	$\log\sigma$	$\log\gamma$	
**Mchoka**	-6.38 [-8.59, -4.18]	-2.88 [-8.66, 2.89]	-5.71 [-7.67 -3.75]	0.445	-7.26 [-12.4, -2.16]	-2.50 [-8.40,3.40]	-3.38 [-8.44, 1.68]	0.0697
**Samama**	-5.44 [-8.64, -2.25]			0.341	-4.80 [-10.8, 1.21]			0.212
**MOET**	-5.40 [-8.71, -2.09]			0.255	-8.26 [-13.4, -3.16]			0.0707
**Koche**	-4.15 [-12.8, 4.46]			0.101	-9.97 [-15.4, -4.57]			0.0256
**St Augustine 2**					-5.73 [-11.1, -0.338]			1.07
**Ndembo**	-5.55 [-8.67, -2.42]			0.185	-4.19 [-11.9, 3.50]			0.618
**Sungusya**	-4.22 [-12.5, 4.07]			0.377	-7.45 [-12.9, -2.04]			1.11
**St Martins**	-4.11 [-13.6, 5.33]			0.423	-9.26 [-14.7, -3.85]			0.0579
**Chikomwe**	-5.57 [-8.66, -2.48]			0.488	-8.05 [-13.3, -2.82]			0.263
**Chipeleka**	-4.23 [-12.5, 4.09]			0.287	-6.73 [-11.9 -1.52]			0.388
**Makumba**	-5.83 [-8.62, -3.04]			0.676	-8.51 [-13.8, -3.26]			0.258
**Mtengeza**	-5.25 [-9.10, -1.40]			0.456	-6.52 [-11.8 -1.29]			0.890

Table S12: Prior *Schistosoma* infection prevalence of $\alpha = 0.20$ for SAC age 6. Parameter estimates for single-β no space model for each species.

Single-β no space with $\alpha = 0.20$

	Biomphalaria AIC=256			MSE	*Bulinus* AIC=NA			MSE
**Parmeters/Schools [CI]**	log β	log σ	log γ		log β	log σ	log γ	
**Mchoka**	-4.96 [-10.1, 0.211]	-3.34 [-8.53, 1.86]	-5.60 [-8.33. -2.86]	0.644	NA	NA	NA	NA
**Samama**				0.341				
**MOET**				0.253				
**Koche**				0.165				
**St Augustine 2**								
**Ndembo**				0.186				
**Sungusya**				0.394				
**St Martins**				0.463				
**Chikomwe**				0.493				
**Chipeleka**				0.334				
**Makumba**				0.682				
**Mtengeza**				0.467				

Confidence intervals

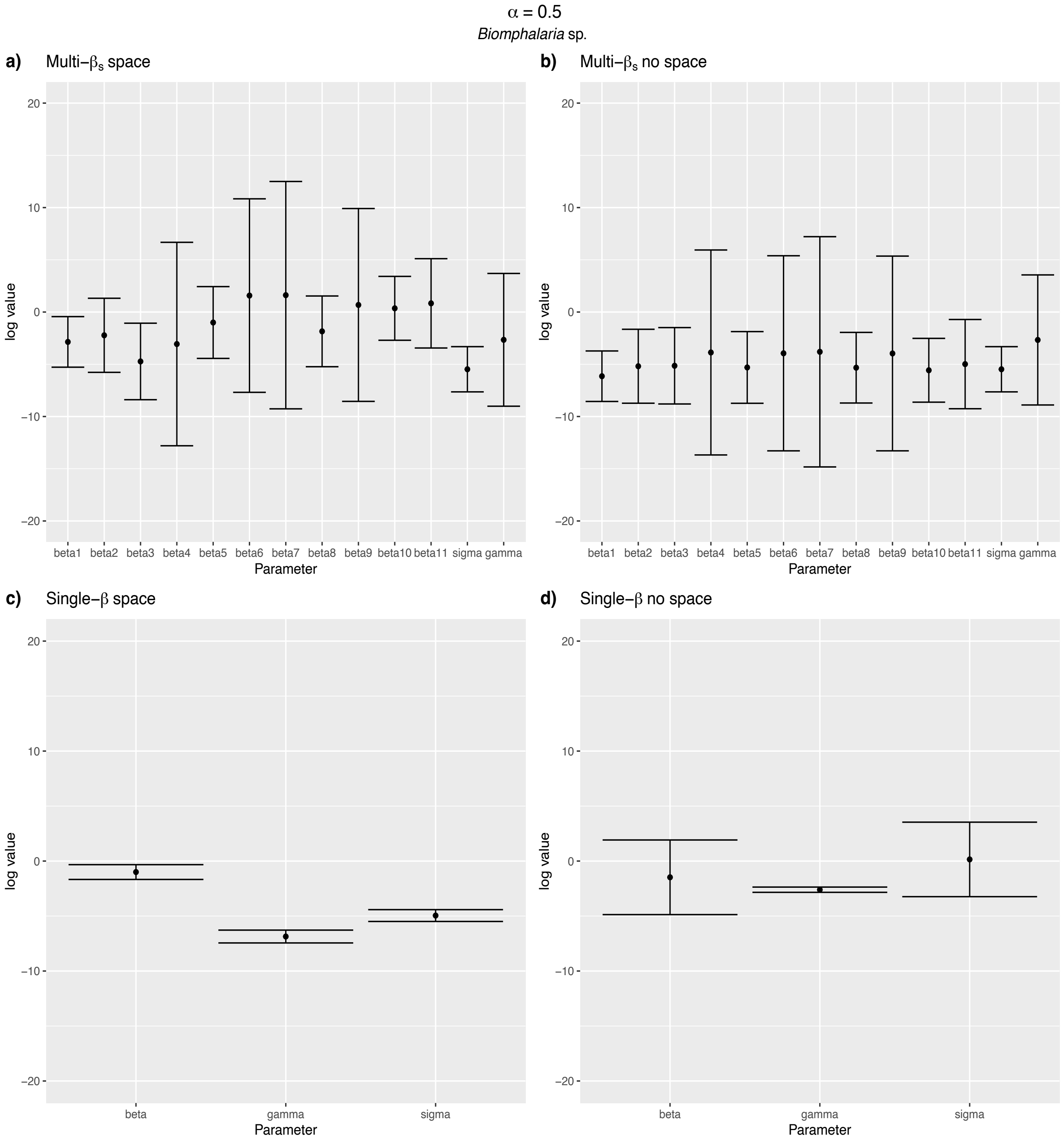

Figure S20: Prior *Schistosoma* infection prevalence of $\alpha = 0.05$ for SAC age 6. Confidence intervals for parameter estimates for *Biomphalaria* sp. models with SAC prevalence at age 6 set as α set to 0.05 (5%) **a)** Multi-β_s space sp. **b)** Multi-β_s no space **c)** Single-β space **d)** Single-β no space

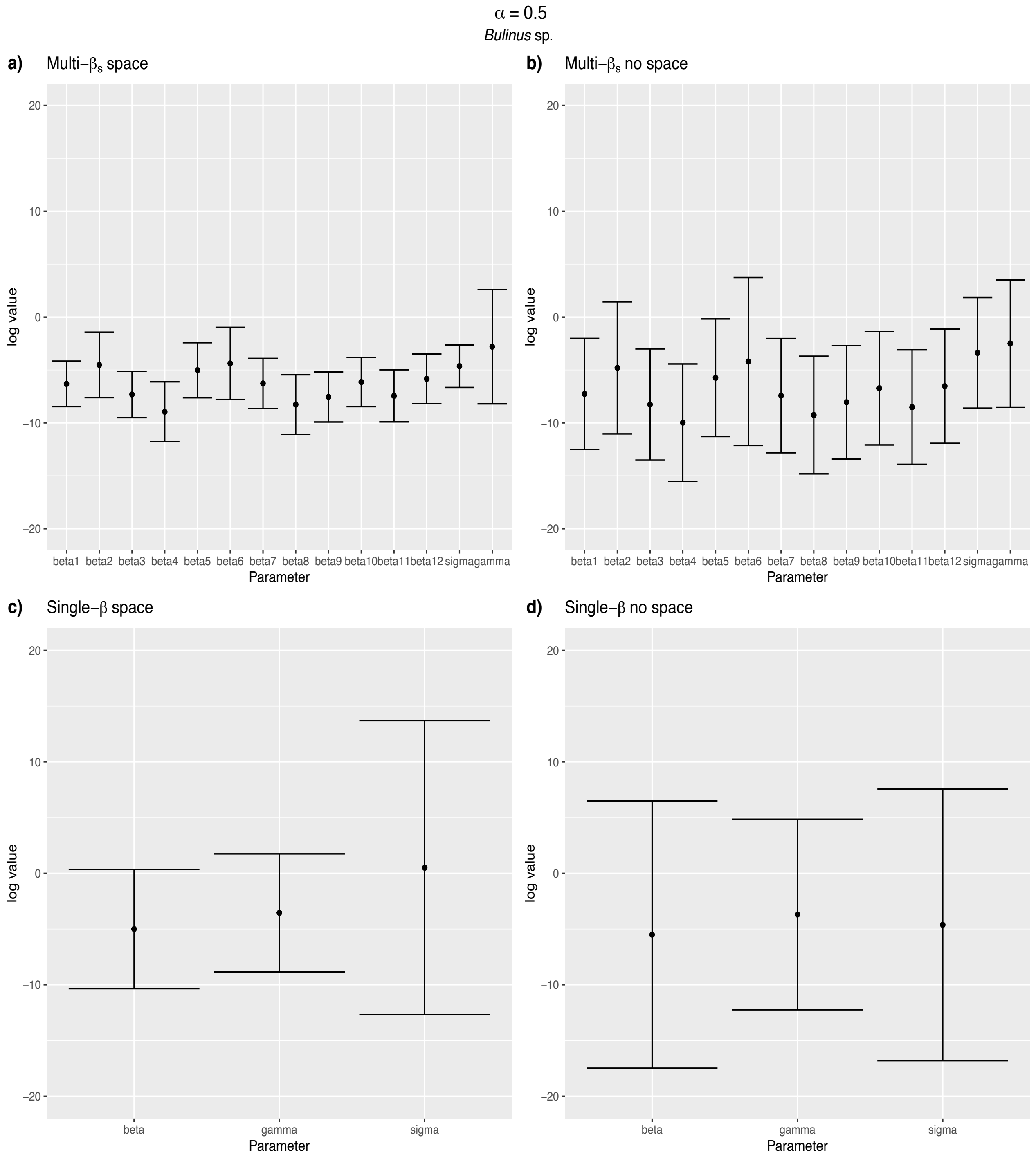

Figure S21: Prior *Schistosoma* infection prevalence of $\alpha = 0.05$ for SAC age 6. Confidence intervals for parameter estimates *Bulinus* spp. models with SAC prevalence at age 6 set as α set to 0.05 (5%) **a)** Multi-β_s space sp. **b)** Multi-β_s no space **c)** Single-β space **d)** Single-β no space.

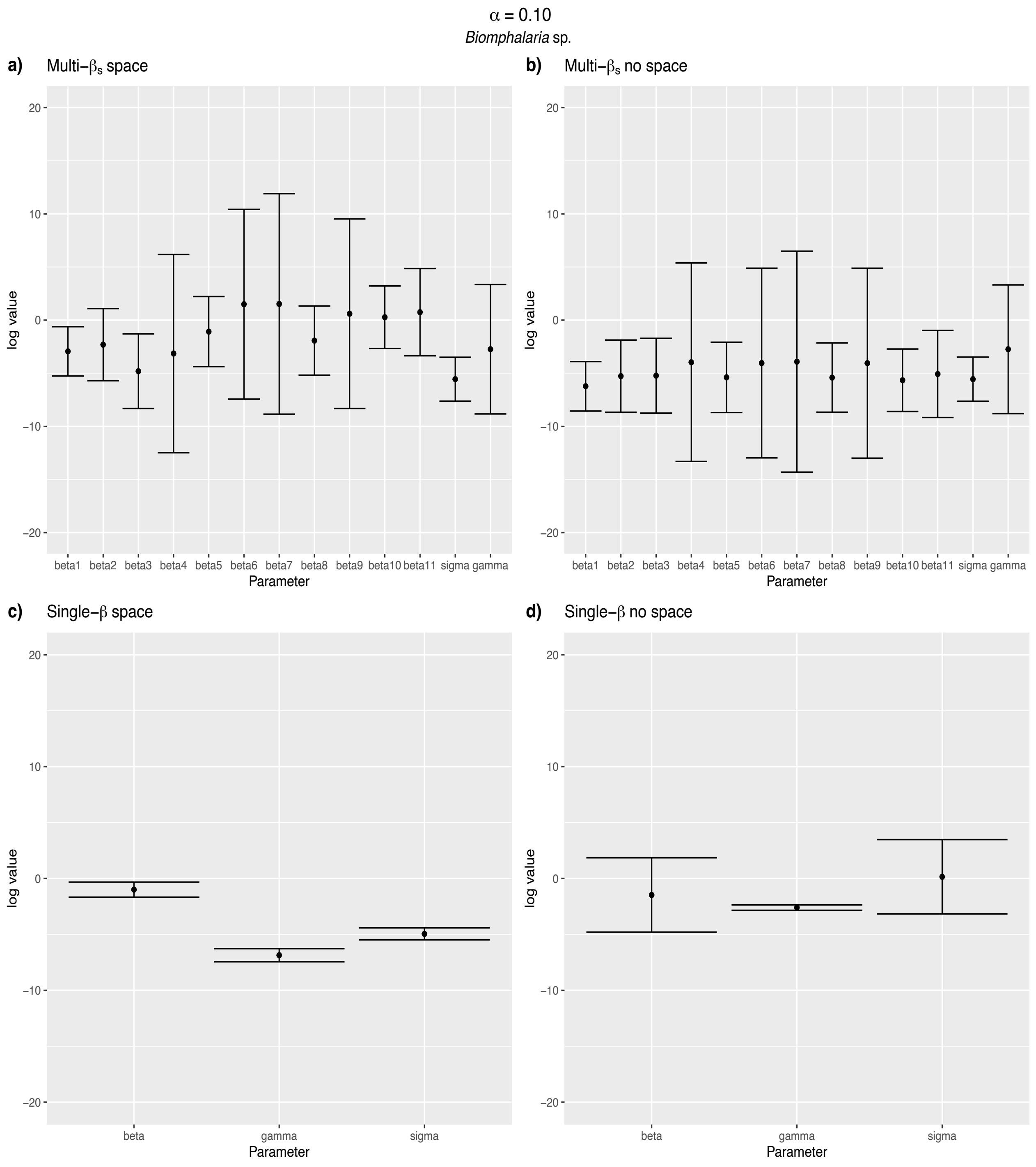

Figure S22: Prior *Schistosoma* infection prevalence of $\alpha = 0.10$ for SAC age 6. Confidence intervals for parameter estimates for *Biomphalaria* sp. models with SAC prevalence at age 6 set as α set to 0.10 (10%); **a)** Multi-β_s space **b)** Multi-β_s no space **c)** Single-β space **d)** Single-β no space.

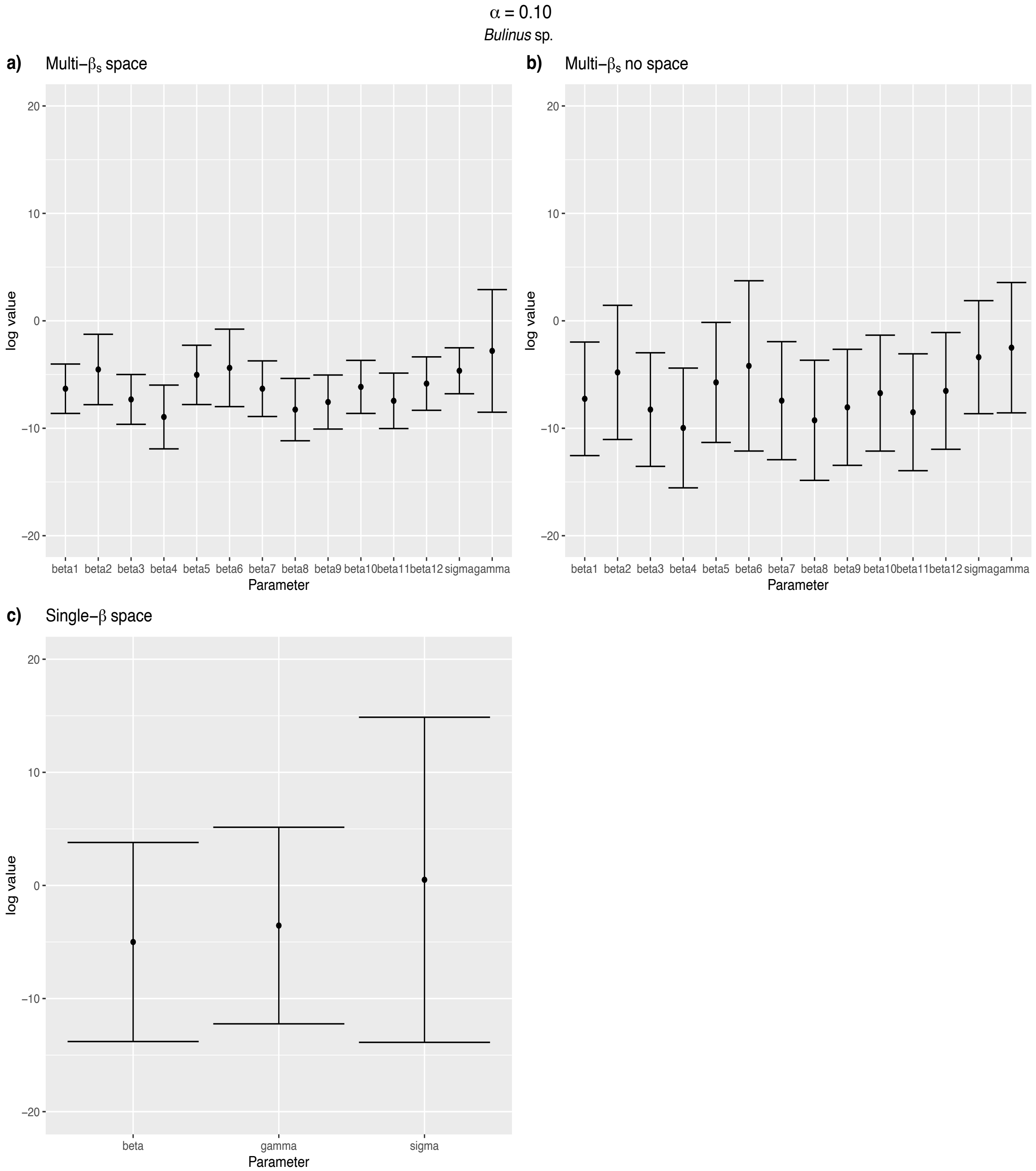

Figure S23: Prior *Schistosoma* infection prevalence of $\alpha = 0.10$ for SAC age 6. Confidence intervals for parameter estimates for *Bulinus* spp. models with SAC prevalence at age 6 set as α set to 0.10 (10%); **a)** Multi-β_s space sp. **b)** Multi-β_s no space **c)** Single-β space

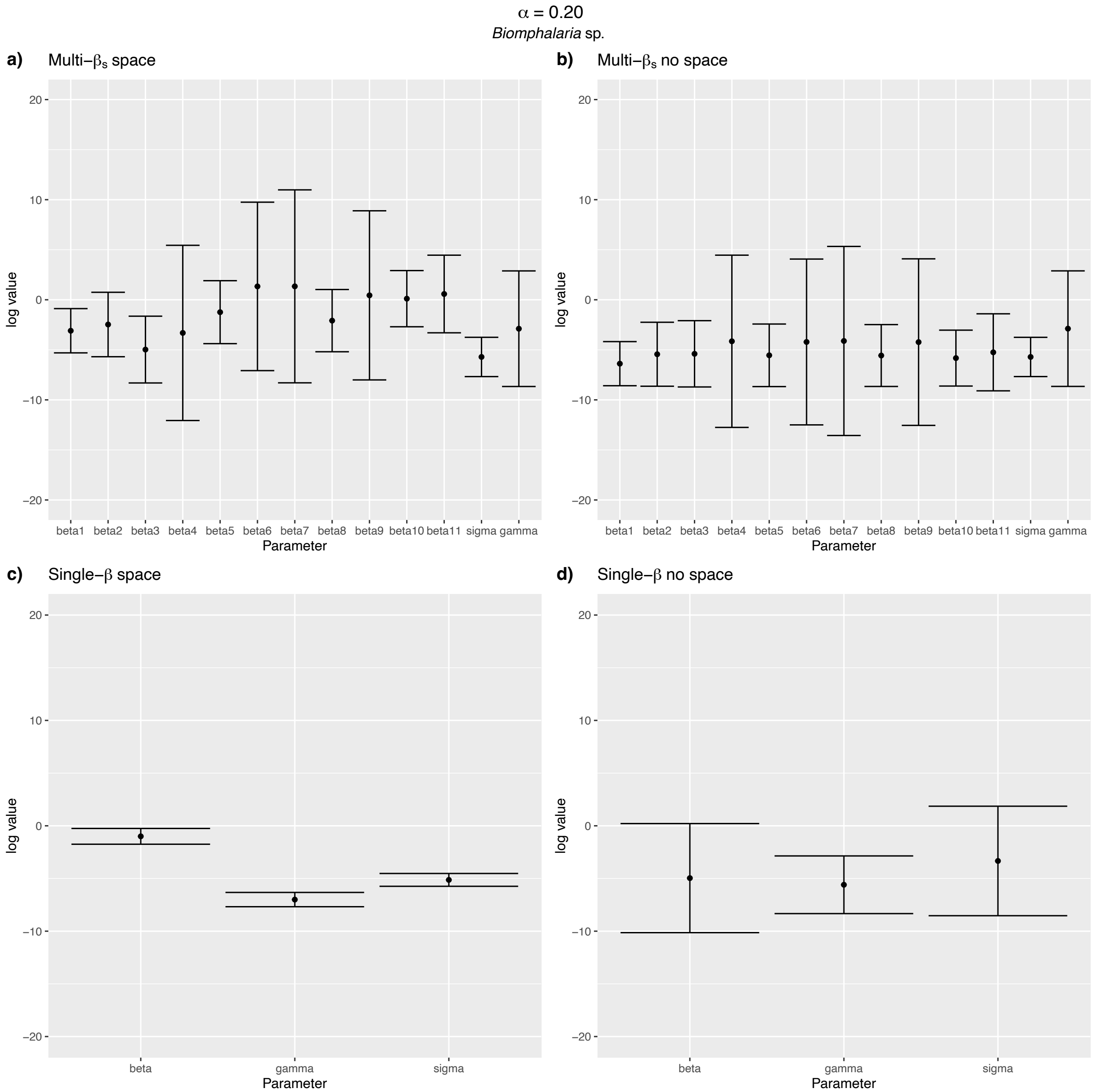

Figure S24: Prior *Schistosoma* infection prevalence of $\alpha = 0.20$ for SAC age 6. Confidence intervals for parameter estimates for *Biomphalaria* sp. models with SAC prevalence at age 6 set as α set to 0.20 (20%): **a)** Multi-β_s space sp. **b)** Multi-β_s no space **c)** Single-β space **d)** Single-β no space

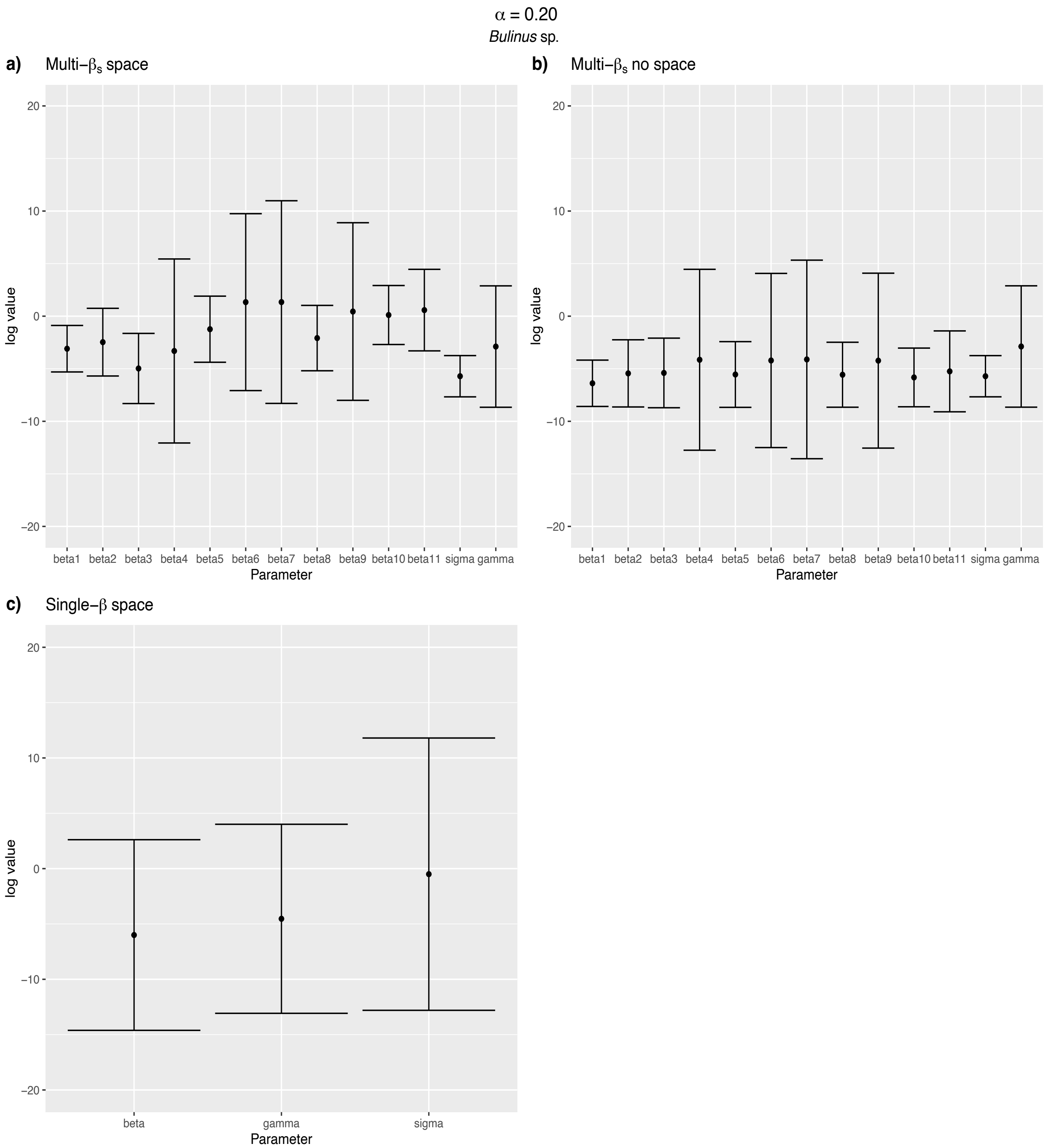

Figure S25: Prior *Schistosoma* infection prevalence of $\alpha = 0.20$ for SAC age 6. Confidence intervals for parameter estimates for *Bulinus* spp. models with SAC prevalence at age 6 set as α set to 0.05 (5%): **a)** Multi-β_s space sp. **b)** Multi-β_s no space **c)** Single-β space
